# The Journey of SCAPs (Stem Cells from Apical Papilla), from Their Native Tissue to Grafting: Impact of Oxygen Concentration

**DOI:** 10.3390/cells11244098

**Published:** 2022-12-16

**Authors:** Marine Mavinga, Mathilde Palmier, Murielle Rémy, Caroline Jeannière, Solène Lenoir, Sylvie Rey, Martine Saint-Marc, Florian Alonso, Elisabeth Génot, Noélie Thébaud, Edith Chevret, Virginie Mournetas, Benoit Rousseau, Claudine Boiziau, Helene Boeuf

**Affiliations:** 1 Univ. Bordeaux, INSERM, BIOTIS, U1026, F-33000 Bordeaux, France; 2 Univ. Bordeaux, INSERM, BRIC, U1312, F-33000 Bordeaux, France; 3 ADLIN Science, Pépinière « Genopole Entreprises », F-91058 Evry, France; 4 Univ. Bordeaux, Animal Facility A2, Service Commun des Animaleries, F-33000 Bordeaux, France

**Keywords:** immunoMap, apical papilla, SCAPs, hypoxia, podosomes, engraftment, thixotropic hydrogel

## Abstract

Tissue engineering strategies aim at characterizing and at optimizing the cellular component that is combined with biomaterials, for improved tissue regeneration. Here, we present the immunoMap of apical papilla, the native tissue from which SCAPs are derived. We characterized stem cell niches that correspond to a minority population of cells expressing Mesenchymal stromal/Stem Cell (CD90, CD105, CD146) and stemness (SSEA4 and CD49f) markers as well as endothelial cell markers (VWF, CD31). Based on the colocalization of TKS5 and cortactin markers, we detected migration-associated organelles, podosomes-like structures, in specific regions and, for the first time, in association with stem cell niches in normal tissue. From six healthy teenager volunteers, each with two teeth, we derived twelve cell banks, isolated and amplified under 21 or 3% O_2_. We confirmed a proliferative advantage of all banks when cultured under 3% versus 21% O_2_. Interestingly, telomerase activity was similar to that of the highly proliferative hiPSC cell line, but unrelated to O_2_ concentration. Finally, SCAPs embedded in a thixotropic hydrogel and implanted subcutaneously in immunodeficient mice were protected from cell death with a slightly greater advantage for cells preconditioned at 3% O_2_.

## 1. Introduction

Mesenchymal stromal/stem cells (MSCs) display a multipotent differentiation capacity and are not subjected to ethical restrictions regarding their clinical use. They have a wide distribution in adult tissues such as bone, adipose tissue and reside in physioxic niches, where O_2_ concentration is much lower than that of ambient air. Amplification of MSCs derived from bone marrow at low oxygen concentration (3% O_2_) stimulates signaling networks involving PI3K/Akt, Notch, and canonical Wnt pathways which contribute to increased self-renewal with decreased differentiation [1,2]. In addition, different studies made in teeth-derived stem cells from human exfoliated deciduous teeth (SHED) have also shown the improvement of bone repair properties of cells after short hypoxic treatment (24 h at 1% O_2_) and/or growth factor priming [3,4]. Human adipose-derived MSCs primed with interferon gamma (IFN-γ) and/or hypoxia treatment display metabolic changes and increased survival by regulating anoikis, apoptosis, and autophagy [5]. MSCs are also found in the apical papilla of the developing tooth root apex and are named SCAPs (Stem Cells from Apical Papilla) [6]. The apical papilla can be obtained from human immature wisdom teeth, which are often extracted in teenagers for orthodontic reasons. SCAPs are a source of highly proliferative and migratory cells endowed with regenerative potential and are able to form dentin in vivo. In addition, these stem cells display dual differentiation potential towards mesoderm and also ectoderm lineages due to their neural crest origin and could be an ideal starting material for obtaining innervated grafts [7,8,9]. The organization of the native tissue from which these adult stem cells are derived is poorly documented. In situ expression of C-X-C chemokine receptor type 4 (CXCR4), the receptor of C-X-C motif chemokine 12 (CXCL12/SDF1a), was shown to colocalize with blood vessel markers like Von Willebrand Factor (VWF) and with stromal 1 (Stro1) and was proposed to be involved in the chemo-attraction of stem cells towards the dental pulp [10]. Also, it was reported that the protease, Fibroblast Activation Protein alpha (FAP alpha), was expressed both in the native tissue and in isolated SCAPs [11]. However, we lack knowledge on the expression of the so-called MSC markers in vivo such as endoglin (CD105 (CD, cluster of differentiation) or Thy-1 (CD90). In addition, during the process of SCAP derivation there is a rupture of the native stem cell niche which affects gene expression as shown with comparative microarray analysis performed with apical papilla and SCAPs, showing, for example, impairment of Bone Morphogenetic Protein 6 (BMP6) expression in SCAPs [12]. We were therefore interested to revisit the expression of SCAP genes or specific adducts in situ, and particularly those recently characterized as being O_2_-sensitive, such as stage-specific embryonic antigen-4 (SSEA4) and the integrin subunit α6 (CD49f) [13,14]. Indeed, SSEA4, a glycosphingolipid, added to proteins or lipids by the beta-galactoside alpha2-3 sialyltransferase (ST3GAL2) enzyme, is present in early embryos, embryonic and adult stem cells [13,15,16] and is considered as a marker of immature cells which decreases with cell differentiation. CD49f, by binding to laminin in basement membranes, is involved in stemness maintenance as shown in both embryonic and adult models. Its deletion leads to devasting skin disease such as epidermolysis bullosa as well as to colitis [17,18,19,20,21]. The dental papilla MSCs develop into dental pulp cells and odontoblasts in vivo [11,22]. The apical papilla is formed at the bud stage as a result of invagination of dental lamina epithelial cells and this tissue is essential for the complete elongation and maturation of teeth roots [23,24]. Podosomes, which are specialized cytoskeletal structures associated with cell migration, could be involved in the remodeling of the apex and teeth root formation [25,26]. For the characterization of the native tissue of SCAPs, it is therefore of great interest to further localize the MSC niches and to highlight the potential presence of podosomal structures in the papilla.

After isolation from the native tissue and during cell culture, SCAPs display a proliferative advantage and a high autophagy flux (an active recycling process), when grown at 3% O_2_ compared with 21% O_2_. While cell morphology is similar at the different O_2_ concentrations at early passages, cells display a more enlarged or elongated phenotype at late passages, at 21% compared with 3% O_2_ ([13] and our unpublished results). We have also observed that their differentiation potentials towards osteogenic and adipogenic lineages diminished over times mainly at 3% O_2_ while chondrogenic differentiation was maintained [13]. The mechanisms involved in the proliferative advantage of SCAPs grown at 3% O_2_ are still unknown. A privileged hypothesis is a differential regulation of telomerase function by oxygen concentration. Indeed, telomere length, which protect chromosome ends from shortening, play essential roles in maintenance of genomic stability. Telomeres shorten over time in somatic cells but not necessarily in stem cells [27,28,29,30]. Telomere length protection is mediated by Telomeric repeat-binding factor 2 (TRF2) whose depletion leads to activation of Zinc Finger And SCAN Domain Containing 4 (Zscan4) in embryonic stem cells. Zscan4 therefore contributes to telomere protection in the absence of TRF2 [31]. The status of telomere length and telomerase activity in SCAPs at different oxygen concentration is still unknown.

Stem cells are influenced by the local environmental physical and biochemical cues within their niches. Hydrogels are attractive materials able to mimic the complex microenvironment of cells and tissues [32,33,34]. The stiffness, viscoelastic properties and topography of the extracellular matrix (ECM) are instrumental in controlling stem cell fate. Natural polymer (chitosan, collagen, alginate)-based hydrogels are promising 3D scaffolds as artificial tissues because of their biocompatibility, biodegradability and tissue-equivalent construction [35,36,37]. A new generation of injectable thixotropic hydrogels, which gel efficiently at 37 °C and re-liquefy upon mechanical constraints, have been recently commercialized. Some of these gels are enriched in ECM proteins or with various types of peptides, enabling specific properties for tissue repair [38,39]. We used one of them, enriched in ECM components, to test the impact of the preconditioning of SCAPs under low O_2_ concentration in a subcutaneous engraftment model [34,40,41,42,43,44,45].

Between the native papilla tissue and the implantation stage, through the in vitro amplification of SCAPs, we aimed at understanding how these MSCs evolve, whether it be their proliferation, the expression of stemness markers or their survival capacity. Therefore, a detailed immunoMap of the apical papilla derived from healthy teeth was performed, with the aim to localize stem cell niches and to determine whether new markers, highly expressed in the derived SCAPs grown under physioxic conditions, are present in the native tissue (e.g., CD49f and SSEA4). In addition, from a tissue engineering perspective using these cells, we also assessed the role of a low oxygen concentration (comparable to the tissue oxygen pressure) on cell properties during the in vitro amplification procedure and their in vivo implantation after seeding in a thixotropic hydrogel scaffold.

## 2. Materials and Methods

The study was conducted in accordance with the Declaration of Helsinki and after approval of the French Research Ministry (DC 2008-412). Wisdom teeth were collected at the Centre Hospitalier Universitaire (CHU) of Bordeaux (Groupe hospitalier Saint André, Bordeaux, France), according to the procedure approved by French regulations. Teeth were collected with informed and oral consent from the donors and of their parents according to the ethical guidelines set by the French law.

### 2.1. Native Tissue Preparation and Cryosectioning

The third molars (wisdom teeth), recovered at the CHU Bordeaux, were directly fixed in ANTIGENFIX (Ref: F/P0016, MM-France, Brignais, France), overnight at 4 °C. The apical papilla was then retrieved shortly in tissue culture hood under sterile conditions and the PBS-washed tissues were incubated in sucrose (15% sucrose in PBS, overnight at 4 °C and then 10 h in 30% sucrose in PBS at 4 °C) before inclusion in OCT (optimal cutting temperature compound, Ref: 12678086, Fisher Scientific, Illkirch Graffensteden, France) at −20 °C. The OCT blocks were kept at −80 °C prior to cryosectioning (10 µm thickness) on a microtome-cryostat (Thermo HM 525 NX, MM France). Then sections, kept at −20 °C, were left 30 min at room temperature (RT), washed in PBS and then stained with Hematoxilin–Erythrosine–Saffron (HES) or immunolabelled with the various primary antibodies. Alternatively, the apical papilla was snap-frozen in OCT. The frozen blocks were kept at −80 °C until cryosectioning as above. Sections 10 µm thick were then fixed for 10 min in ANTIGENFIX and processed as above. Both procedures were efficient and gave similar results.

### 2.2. Staining and Immunolabelling of Tissue Sections

HES staining: tissue sections from apical papilla, and mouse skin after explantation, kept at −20 °C, were left 30 min at RT, washed in PBS and stained with HES (Hemalun, Ref: 1.09249.2500, Saffron, Ref: 11507737_27481.105, Erythrosine-239, 720-0179, in VWR, Fontenay-sous-Bois, France) to check the quality of the sections concerning cells and extracellular matrix organization.

Immunohistochemistry: fixed sections were immunolabelled with various antibodies following a previously established procedure [46]. Briefly, papilla sections were permeabilized with PBS containing 0.3% Triton X-100 for 15 min at RT, then blocked in PBS containing 0.1% BSA, 10% normal goat serum, 0.2% Triton X-100, and 0.05% Tween-20 for 1 h at RT. Primary antibodies were added in the blocking solution overnight at 37 °C and after 2 washes for 5 min at 37 °C in PBS containing 0.05% Tween-20, the secondary antibodies were added in the blocking solution, 2 h at 37 °C. After two washes for 5 min in PBS containing 0.05% Tween-20, nuclei were stained with DAPI in PBS for 5 min at RT. Then, Fluoromount-G Mounting Medium was added (Fisher Scientific, ref: 15586276) and sections were mounted with cover slips and sealed with nail polish. Images were captured on a confocal microscope (Leica TCS-SPE, Nanterre, France).

Primary antibodies: Oct4: ab19857; Sox2: ab97959; Nanog: ab109250, all from Abcam, Amsterdam, Netherlands diluted 1/200; SSEA4: MC 813-70 (InvitroGen, Fisher Scientific), 1:100; CD49f: BTMC-A145-7GA (Biotrend, Clinisciences, Nanterre, France), 1: 200; CD105: 14-1057-82 (InvitroGen), 1:200; CD90: 14-0909-82 (InvitroGen), 1: 200; CD31/PECAM1: 14-0319-82 (InvitroGen), 1: 250 or CD31/PECAM1 from Santa Cruz, Clinisciences, Nanterre, France: 53411, dilution 1: 100; Von Willebrand factor: A0082 (DAKO, Agilent Technologies, Les Ulis, France), 1:200; Tks5/SH3PXD2A (SH3 and PX domains 2A): novus NBP1-90455 rabbit: 1:100; cortactin 4F11 clone: 05-180 Merck/Sigma Millipore, Molsheim, France, 1:100; Tomm20: 11802-1-AP 1:200 (Proteintech, Fisher Scientific); mitofilin recombinant [EPR8749] Abcam,: ab137057; Ki-67 conjugated with Alexa Fluor 488 [clone B56], BD/Pharmingen: 561165; Control isotype Alexa 488, BD/Pharmingen: 557782. The secondary antibodies, 1:1000 were from InvitroGen: Goat anti-rabbit conjugated to Alexa Fluor 568 (A-11036), or to Alexa Fluor 488 (A-11008); Goat anti-mouse conjugated to Alexa 568 (A-11031), or to Alexa Fluor 488 (A-11001), depending of the primary antibody used.

### 2.3. Establishment of SCAP Banks, Cell Culture and Growth Curve Analysis

All SCAP derivation and cell cultures were performed as previously published [13], using alpha MEM (Minimum Essential Medium) (Gibco, Fisher Scientific, A10490-01) culture medium supplemented with 10% fetal bovine serum (FBS, D. Dutscher, Bernolsheim, France, batch N°: S00CH10104, France) and gentamycin (Gibco, 15750-037, 40 µg/mL). The growth curves were established by direct cell seeding and counting as previously described [13].

The new banks derived from donors 4, 5 and 6 were named: UBx-SCAP-N4, N5, N6 (derived and amplified at 21% O_2_) or UBx-SCAP-H4, H5, H6 (derived and amplified at 3% O_2_), according to the previous published banks [13]. Cells were stored for a few months at −80 °C or in liquid nitrogen for years. A human induced Pluripotent Stem Cells (hiPSC) line, derived from the IMR90 fibroblasts, was previously described [47].

The 3% O_2_ concentration was chosen based on previous experiments performed with murine embryonic stem cells (mESCs) which maintain their stemness under this condition [48]. We carried on experiments on SCAPs at this potential pertinent O_2_ concentration which could correspond to physioxic O_2_ concentration in wisdom teeth.

### 2.4. Flow Cytometry

Expression of CD49f and SSEA4 was quantified by flow cytometry as already published [13].

### 2.5. Telomere Length and Telomerase Activity

Telomere length (TL) was calculated by means of Absolute Human Telomere Length Quantification qPCR Assay Kit according to the manufacturer’s instructions (CliniSciences), as already published [49]. Telomerase activity (TA) was assessed from protein extracts using the TRAP assay (TRAPeze telomerase detection kit; S7700, Millipore) as previously described [50]. Both TL and TA experiments were carried out on a Stratagene Mx3005P system and analyzed with MxPro 4.01 QPCR software Stratagene (both from Agilent Technologies, Les Ulis, France). Each sample was run in duplicate with control DNA.

### 2.6. Lentiviral Infection and Engraftment in NSG Mice

The authorization of animal experimentation is APAFIS #31524-2021042810216303 v3 and the agreement number is B33063916. UBx-SCAP-N1 and UBx-SCAP-H1 (250,000 cells) at passage 4 were transduced with the lentivirus encoding the luciferase gene (from the Flash Therapeutics/Vectalys company, Toulouse, France): ILV-EF1a-LUC-9-001 (titer: 2.5 × 10^9^/mL) at a multiplicity of infection (MOI) of 10. Ten days after the infection, cells were plated on 96 wells plates, grown for 2 days and incubated with 3 mg/mL luciferine, in 100 µL, then analyzed with the photon imager (Biospace Lab, Nesles-la-Vallée, France). The minimum sensitivity of SCAPs-Luc^+^ detection was 300 cells, giving a photon/second/steradian (ph/s/sr) value 6 times higher than the background (our unpublished data). Cells were amplified and at passage 7, 200 µL of a cell suspension (at 9 million cells/mL in alpha MEM medium + 30% FBS without antibiotics) was mixed with 400 µL of the vitroGel hydrogel matrix (Well bioscience, TebuBio, Le Perray-en-Yvelines, France, VHM01). The mixture was included in a 1 mL syringe, and capped (to avoid the oxygenation of solution prepared with cells at 3% O_2_). Needles of 26G were used for the sub-cutaneaous injection. For cells injected without hydrogels, tubes containing 3 million cells/mL in alpha MEM medium containing 10% FBS (w/o antibiotics) were prepared. The syringes and tubes were brought in the A2 animal faculity. A total of 500 µL of each solution were injected subcutaneously in NSG mice (10 weeks old, female, NOD.Cg-Prkdcscid Il2rgtm1Wjl/SzJ), 10 mice per condition. A similar cell quantity (1.5 million with or without hydrogel) was then injected.

At different time points (Days 1, 4, 7, 11, 14, 17, 21, 28 and 44) the luciferase activity was assessed after intraperitoneal injection of 100 µL luciferine (30 mg/mL). The values, for all the analysis, were taken at the activity plateau. At day 23, 3 mice of each condition were sacrificed for histology analysis and at day 44 all the remaining mice were sacrificed. The grafts with skin were recovered and incubated immediately in ANTIGENFIX for further analyses.

### 2.7. Analysis of the Grafts

Grafts from the mice, fixed for 4 h in ANTIGENFIX at room temperature, were washed twice with PBS and incubated overnight in PBS containing 15% sucrose at RT and then for about 10 h in PBS containing 30% sucrose. The samples were then embedded in OCT, 30 min at −20 °C and then kept at −80 °C until sectioning (10 µm sections) with the cryostat. Immunolabelling was performed with the same procedure as for the sections of apical papilla.

### 2.8. Statistical Analysis

Statistical analyses for the in vivo experiments were conducted using jamovi (The jamovi project (2021). jamovi (Version 1.6) [Computer Software]. Retrieved from https://www.jamovi.org, software uploaded in September 2020, last analysis in September 2022). All performed tests were non parametric and are specified in the figure legends. Briefly, either Wilcoxon signed-rank tests (multiple groups) or Mann-Whitney U tests (two groups) were performed (***: *p* value < 0.001; **: *p* value < 0.01; *: *p* value < 0.05).

## 3. Results

### 3.1. ImmunoMap of Native Apical Papilla Tissue Derived from Healthy Wisdom Teeth of Teenagers

**Figure 1:**
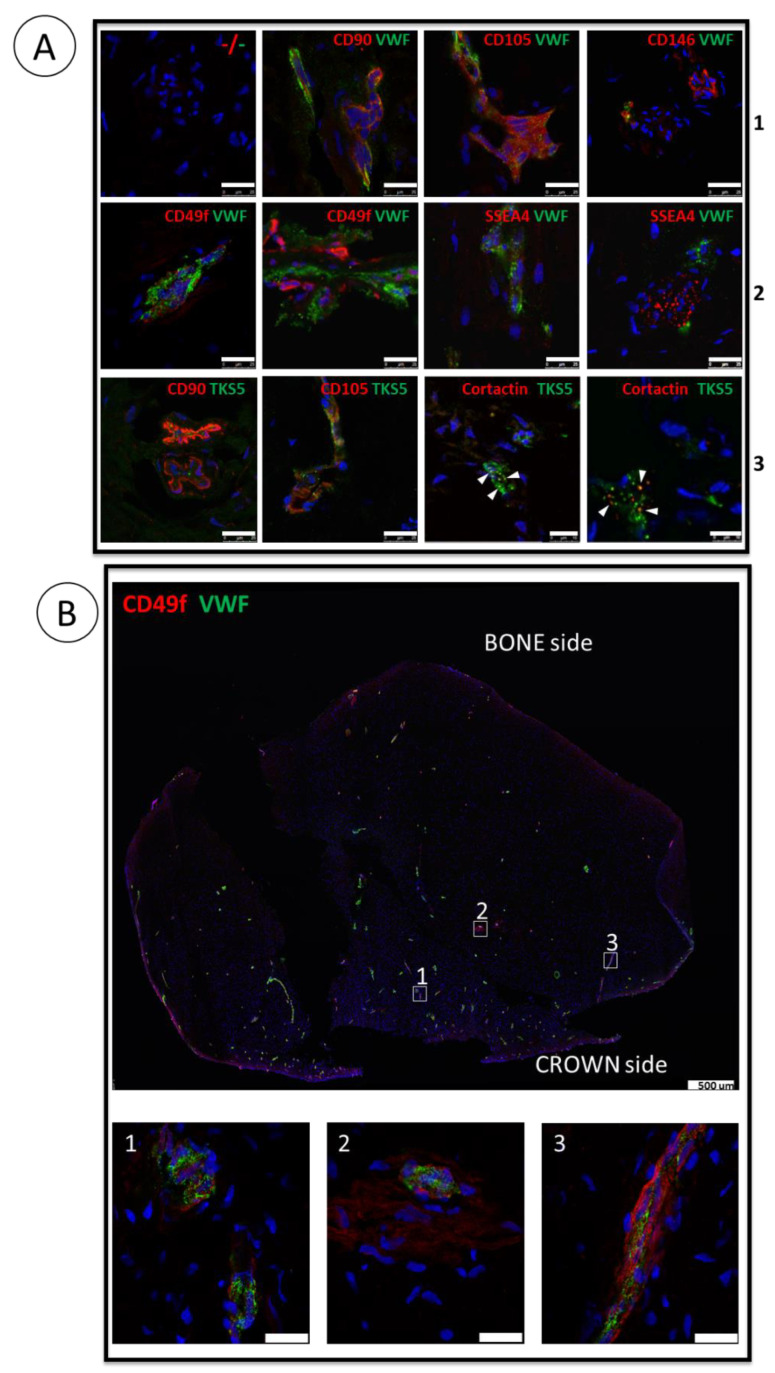
ImmunoMap of the native apical papilla tissue. (**A**) Immunolabelling of 10 µm sections of apical papilla tissue with the indicated antibodies. Nuclei were counterstained with DAPI. Markers analyzed (red/green): **1**: MSC/blood vessels; **2**: Stemness/blood vessels; **3**: Podosome-like structures. Scale bar is 25 µm except for double labelling Cortactin/TKS5: 10 µm. White arrows indicate the colocalization of Tks5 and cortactin, a hallmark of podosome-like structures. (**B**) Immunolabelling of an entire longitudinal section with CD49f and VWF and enlargement of the boxed areas. Scale bar is 500 µm for the entire section and 25 µm in the enlarged images.

Sections of apical papilla from five independent teenagers were analyzed with various antibodies alone or in combination, aiming to characterize the markers of interest in situ, some of them being expressed by SCAPs established in vitro. First, we stained the tissue with Hematoxylin–Erythrosine–Saffron (HES) to verify the section quality regarding cells and ECM organization which were optimum with many collagen rich blood vessels easily identified (Figure S1A). As a positive control of the immunostaining procedure, we detected a broad expression of Translocase of the outer mitochondrial membrane 20 (Tomm20), a specific mitochondrial marker, on the section (Figure S1A). In sections analyzed from the different donors, we performed CD31/platelet and endothelial cell adhesion molecule 1 (PECAM-1) or Von Willebrand Factor (VWF) staining to detect blood vessels. We found them scattered throughout the papilla and detected the classical MSC markers CD105, CD146 and CD90 (Figure 1A and Figure S1A). The stemness markers CD49f and SSEA4, which are highly expressed in SCAPs cultured under 3% O_2_ [13]_,_ were detected at the periphery of the blood vessels but also in areas away from the vessels (Figure 1A). Analysis of the entire section for the expression of CD49f and VWF (Figure 1B) or for CD31, or CD105 (Figure S1B,C) revealed the overall vasculature of the sections and the expression of CD49f in the vicinity, and also away from the vessels. Figure S1D shows the negative control (omitting the primary antibody) of the entire section. In addition, analysis of adjacent sections of papilla, immunolabeled with each antibody (CD105, CD146, CD90 CD49f and SSEA4) and with VWF highlights common areas of co-staining and shows also specific areas of CD49f and SSEA4 staining away from vessels and from MSC markers (Figure S2A–F). These results better characterize the stem cell niches of the apical papilla, showing that they are not only perivascular but may also reside away from blood vessels. In addition, embryonic stem cell markers such as Octamer-binding transcription factor 4 (Oct4), SRY-Box Transcription Factor 2 (Sox2) or Nanog were not detectable in the native tissue (data not shown). Since apical papilla is a structure involved in the edification of the dental roots, we seek for organelles associated with cell migration. We investigated the expression of the podosomal markers Tyrosine Kinase Substrate with 5 SH3 domains (Tks5) and cortactin to detect the cytoskeletal microdomains [25,51]. Podosome-like structures were indeed detected in some areas of apical papilla, close to blood vessels and/or MSC-containing areas or at the periphery of the sections (Figure 1A and Figure S1A,E). The colocalization of Tks5 and Cortactin (yellow dots in Figure 1A and Figure S1E), highlighted the presence of podosome-like structures in this tissue. Last but not least we detected very few cells expressing Ki-67, a proliferation marker highly expressed in cultured SCAPs, indicating that cells are not extensively dividing in the apical papilla tissue (Figure S1A).

This study provides, for the first time, an immunoMap of the apical papilla with localization of stem cell niches and of podosome-like structures and establishes that CD49f and SSEA4, known to be expressed by SCAPs in physioxic conditions in vitro, are indeed expressed in the tissue of origin in vivo. However, the markers were expressed similarly in the different areas of the papilla (bone or crown sides), indicating that there is no obvious regionalization of expression of the selected markers.

### 3.2. Proliferative Advantage of SCAPs Banks Derived at 3% O_2_ in Comparison with 21% O_2_ and Stability of SCAPs Banks over Time

**Figure 2:**
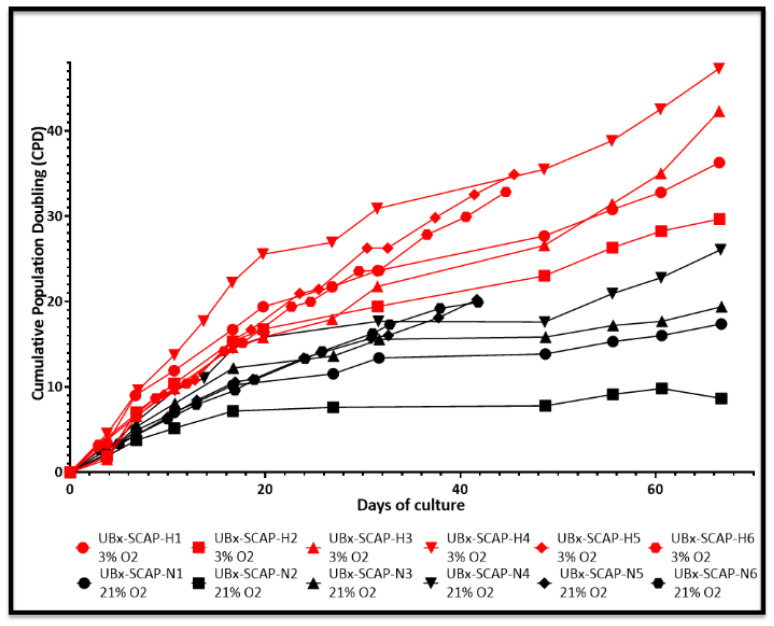
Proliferative advantage of SCAPs banks derived at 3% O_2_ in comparison with 21% O_2_. Curves passing by all points represent the cumulative population doubling of SCAPs grown for the indicated number of days.

**Figure 3:**
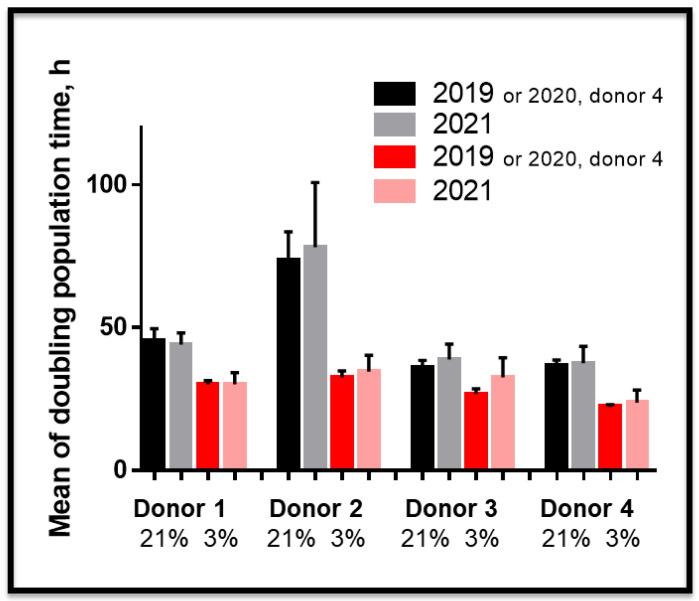
Stability of SCAPs banks over time. Mean + SEM of doubling population time of SCAPs, for the first 10 passages performed at different times as indicated after freeze/thaw processes. All banks, derived and frozen in 2018 (donors 1, 2, 3) or 2020 (donor 4) were frozen/thawed several times (at least 3 times) and then followed for the first 10 passages by thawing passage 1 of each bank.

We next explored the effect of O_2_ concentration on SCAP amplification in vitro. Six banks were derived using an established procedure [13]. We then followed the growth curve of these new banks (named UBx-SCAP-N4-6 (derived and amplified at 21% O_2_, black curves) or UBx-SCAP-H4-6 (derived and amplified at 3% O_2_ red curves)) along with those previously reported (N1-3 and H1-3), [13], for 45 to 65 days (Figure 2). Growing cells at low O_2_ concentration conferred a proliferative advantage. In addition, low O_2_ concentration enabled cells to be grown for longer period of time (more passages) without entering in senescence (Figure 2 and data not shown). Heterogeneity was noticed between the six 13–15-year-old healthy donors. In addition, we investigated the stability of these banks over time and showed that after several freeze/thaw cycles (at least three cycles), the proliferative advantage was kept (Figure 3) along with the high expression of CD49f and SSEA4 markers at 3% O_2_ (Figure S2).

### 3.3. Telomere Length and Telomerase Activity in SCAPs Are Not Critically Regulated by Oxygen Concentration

**Figure 4:**
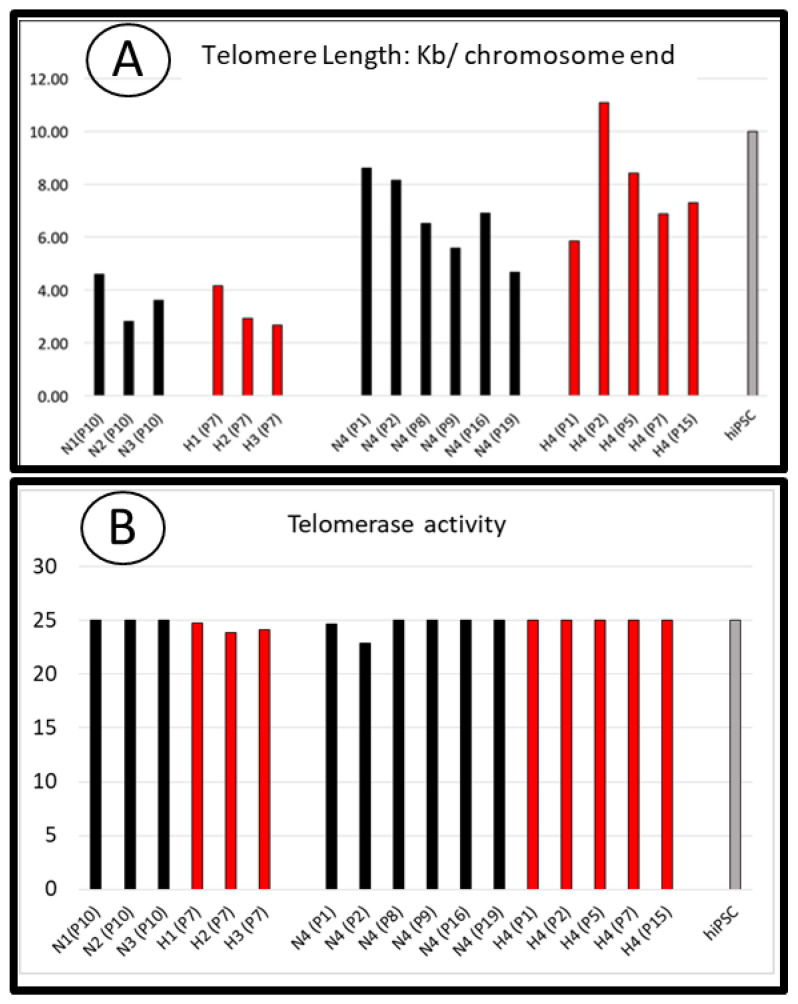
Telomere length and telomerase activity in SCAPs grown at different O_2_ concentrations. (**A**) Telomere length of SCAPs from three donors (N1 to N3) at passages (P) which correspond to similar cumulative population doubling (25 DPC) and for the donor 4 at different passages as indicated, N (21% O_2_) and H (3% O_2_). The hiPSC cell line (established from IMR90 fibroblasts, Passage 35) is included as a positive control of a highly proliferative cell line. (**B**) Telomerase activity on same cell samples as in A).

We investigated the mechanisms underlying the proliferative advantage of SCAP banks grown at 3% O_2_ versus 21% O_2_ and those involved in a greater expansion potential over time. Since we did not detect critical variations in the cell cycle phases of SCAPs grown at 3% or 21% O_2_ ([13] and data not shown), we tested the hypothesis that telomerase activity and therefore telomere length might be regulated differently depending on O_2_ concentrations. We analyzed these parameters in SCAPs grown for several periods of time under both conditions. Each sample was analyzed for both telomere length and telomerase activity (Figure 4A,B). As telomere shortening is associated with cell divisions, telomere lengths were analyzed after the same number of cell divisions rather than at the same number of passages.

For donors 1 to 3, we assessed telomere length of cells which had divided 25 times (Figure 4A). For donor 4, we performed the analysis at different passages, some of them corresponding to similar rounds of division: for instance, SCAPs-N4 at passage 8 (N4, P8) is comparable, in term of cell division number, to H4 at passage 5 (H4, P5). We did not observe significant differences in telomere length along with similar telomerase activity at either O_2_ concentrations (Figure 4A,B). Telomerase activity was slightly higher at 3% O_2_ in some experiments (data not shown), but this could not explain the greater long term proliferative potential of SCAPs under this condition. Interestingly, telomerase activity of SCAPs was similar to that of highly proliferative hiPSC cells. However, this activity alone could not account for the observed variation in telomere lengths, indicating that other mechanisms of telomere maintenance might be operational and regulated in adult stem cells.

### 3.4. Implantation: Behavior of SCAPs Embedded within Thixotropic Biomaterials

**Figure 5:**
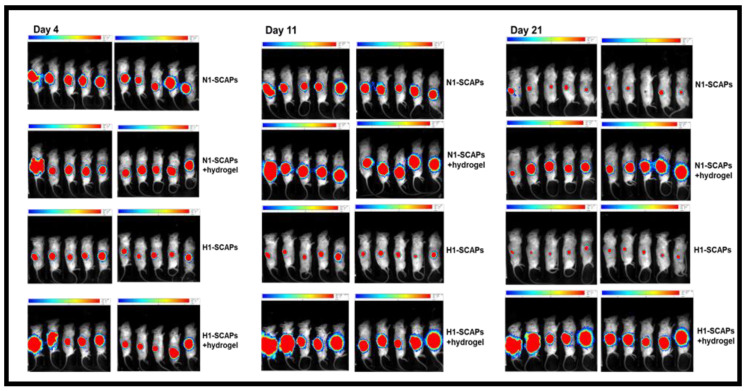
Bioluminescence imaging of mice analyzed 4, 11 or 21 days after sub-cutaneous injection of SCAPs. The quantity of surviving SCAPs-Luc^+^ from donor 1 grown at 21% (N1-SCAPs) or 3% O_2_ (H1-SCAPs), and injected alone or included in the hydrogel was measured by luminescence. It was measured for 3 min at the same gain for all mice (n = 10/condition). The Luc measurements were used to draw graphs shown in Figure 6.

**Figure 6:**
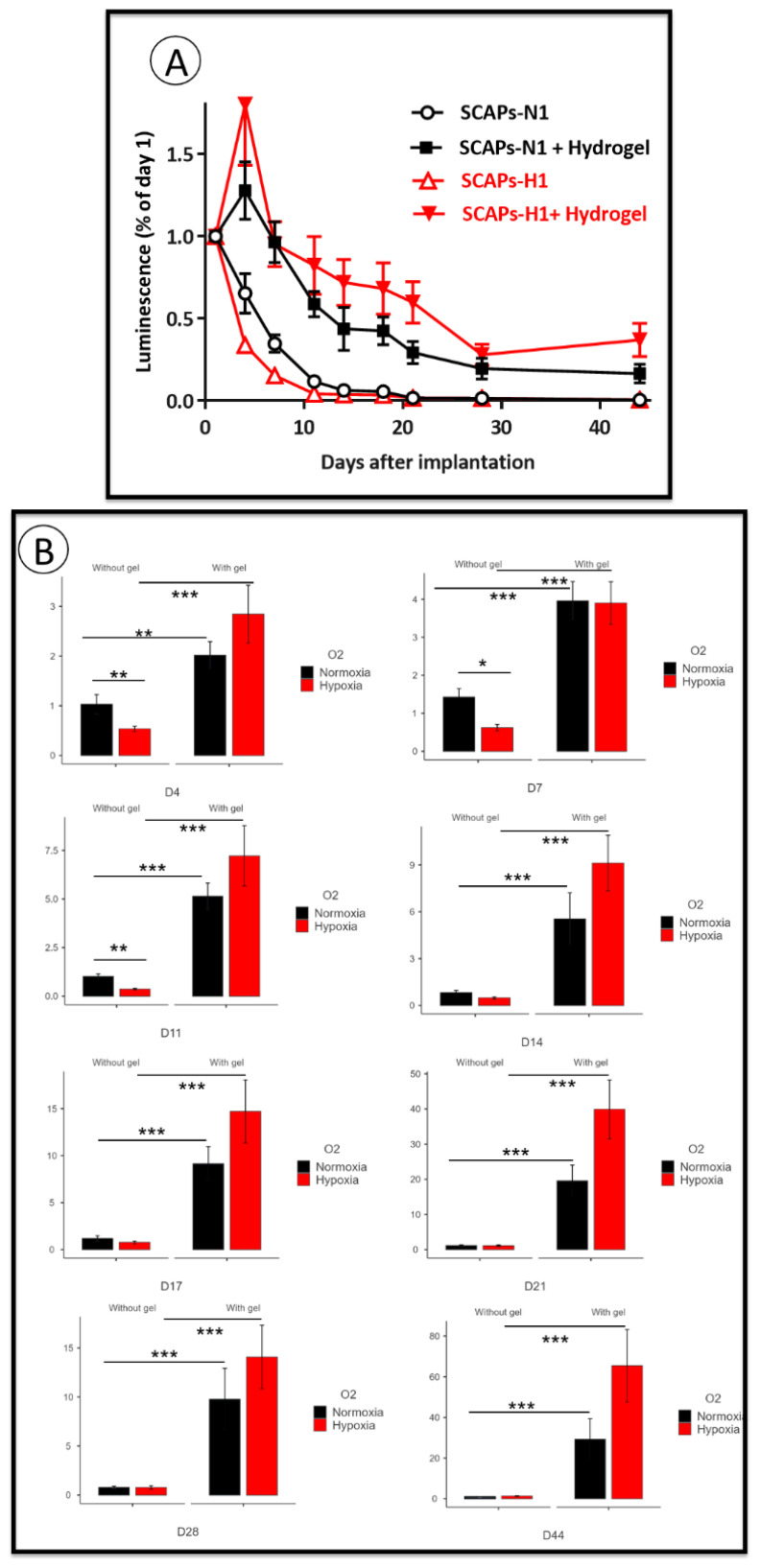

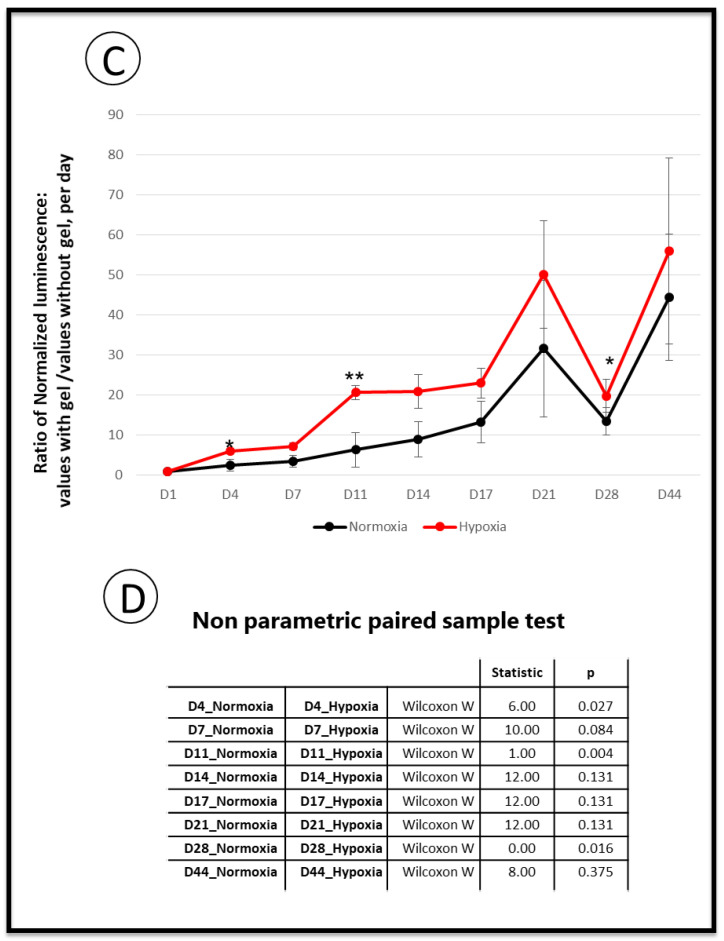
SCAPs survival after implantation. Luminescence measured in mice after subcutaneous implantation of SCAPs-Luc^+^ preconditioned at 3% O_2_, or 21% O_2_ and seeded or not in the hydrogel. (**A**) Graphs of the mean + SEM of the decrease of luciferase (Luc) activity over time, of the 4 groups of mice injected with SCAPs alone at 21% O_2_ (SCAPs-N1) or at 3% O_2_ (SCAPs-H1) or with cellularized hydrogels with cells at 21% (SCAPs-N1 + Hydrogel) or at 3% O_2_ (SCAPs-H1 + Hydrogel). All data were calibrated with the first measure of Luc arbitrarily put at 1 (which represents the 100% of luminescence). Detailed data with statistical analysis are in Supplementary Figure S3. (**B**) Graphs of luminescence intensity, normalized for each day with the same mice taken at 1. (Mann–Whitney U, ***: *p* value < 0.001; **: *p* value < 0.01; *: *p* value < 0.05). (**C**) Curves representing the ratio of normalized luminescence of SCAPs-Luc^+^ cells with or without gel as followed: [Cells with hydrogel/cells alone (H, 3% O_2_)] over [cells with hydrogel/cells alone (N, 21% O_2_), measured at the different days in the engrafted mice, as indicated in the two conditions. Normoxia: 21% O_2_ or Hypoxia: 3% O_2_. (**D**), Statistical analysis of the calculated ratio at each day (Wilcoxon signed-rank test, **: *p* value < 0.01; *: *p* value < 0.05), shown on (**C**).

**Figure 7:**
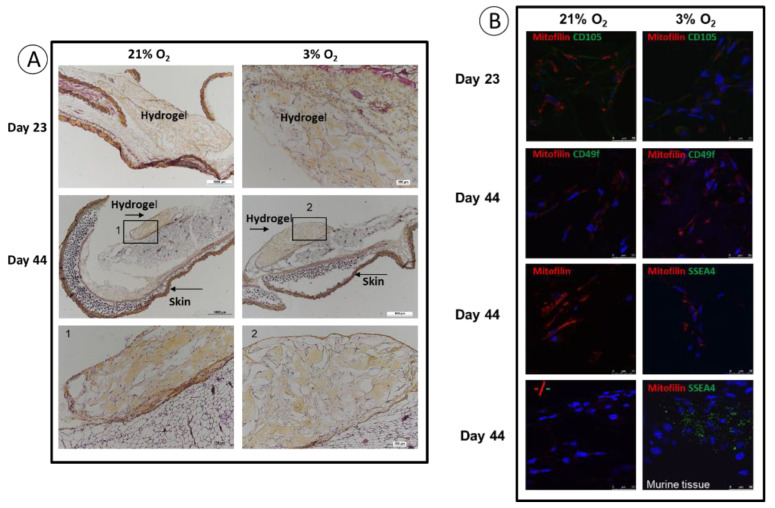
Representative pictures of HES and immunolabelling of sections of grafts recovered at day 23 or day 44. Representative sections of grafts in mice that received SCAPs-Luc+ amplified at 21% or 3% O_2_ and seeded in the hydrogel. (**A**) HES staining, with enlargement of hydrogel parts as indicated. (**B**) Immunolabelling with the indicated antibodies. Nuclei are stained with DAPI. The anti-SSEA4 antibody which recognizes the human and mouse cell surface antigen was not expressed along with mitofilin, but was detected in mouse tissue as indicated. Scale bar is 25 µm for panel B.

A major goal in stem cell research is to optimize the conditions for cellularized biomaterial implantation, for tissue repair towards clinical applications. We have set up a procedure in an animal model in which a commercial thixotropic hydrogel (VitroGel Matrix) is mixed with SCAPs preconditioned at either 21% or at 3% O_2_. SCAP banks N1 and H1, which display, as the other banks, an important difference in cell proliferation at 21% compared to 3% O_2_ (Figure 2), were transduced with lentivirus expressing luciferase (SCAPs-N1-Luc^+^ and SCAPs-H1-Luc^+^). Cells either alone or in combination with the hydrogel, were injected subcutaneously in immunodeficient NSG mice. The quantification of luciferase activity, assessing cell survival, was performed regularly over a 44 days period. Figure 5 shows images of the mice with the injected SCAPs-Luc^+^ at different time points. Survival of the SCAPs-Luc^+^ decreased over time within the first two weeks when injected alone and survived unexpectedly better, up to day 11, when grown at 21% O_2_, than at 3% O_2_ (Figure 6A,B and Figure S3). A higher survival rate was observed with the cells injected in association with the hydrogel (Figure 6A,B), but no statistical difference was obtained between the two oxygen concentrations when pre-seeded in the thixotropic hydrogel. However, we noticed that the protective effect of the hydrogel was greater for cells preconditioned at 3% O_2_ when comparing the ratio of luminescence of cells with or without hydrogel at 21% or 3% O_2_ (Figure 6C,D). We then characterized the cellularized grafts recovered from mice after two time periods at day 23 (three mice per condition were sacrificed) and at day 44, (final point of the experiment). HES staining allowed to determine the presence of the hydrogel in the sections and we could detect it in all the analyzed mice up to 44 days. This indicates a good biocompatibility of the hydrogel well supported by the animals. In addition, we could easily detect the presence of cells in the hydrogel after both 23 and 44 days (Figure 7A). Immunolabelling of sections with human mitofilin (a mitochondrial protein with an antibody specific of the human protein), with CD105, CD49f, SSEA4, and Ki-67 demonstrated that all cells present in the hydrogel were of human origin (Figure 7B). In contrast, neither MSC markers (CD90 and CD105) nor SSEA4 or CD49f were detected in the mitofilin positive areas, indicating that SCAPs-Luc^+^ cells, within the mice skin, could survive and had lost their MSC phenotype (Figure 7B and data not shown). The only labelling with SSEA4 was found in some areas of mice tissues that we did not characterize further. In addition, we could not detect dividing cells (no Ki-67 positive cells) indicating that the surviving cells were quiescent in the graft after 44 days. These results showed that VitroGel matrix represents a suitable easily injectable cell support that ensures cell survival. However, conditions for better long-term survival of SCAPs can still be improved.

## 4. Discussion

The optimization of isolation, amplification and long term conservation of therapeutical adult stem cells and the selection of appropriate scaffolds for engraftment in diseased tissues remain a challenge for cellular therapy and tissue reconstruction [52,53]. Microenvironment parameters like the oxygen concentration which is low in stem cell niches (1% to 5%), should also be taken under consideration for clinical applications. Knowledge of the organization and composition of the native tissue from which adult stem cells are derived will also give cues regarding the essential features of stem cell niches.

The apical papilla tissue is a vascularized cushion that lies between the dental crown and the teeth roots in formation. The immunomapping of this tissue, presented in this report, highlights specific blood vessel-rich areas, perivascular regions but also regions remote from blood vessels enriched with cells expressing the embryonic and adult stem cell markers CD49f and SSEA4 [15,16]. We therefore provide a marker-based localization of stem cell niches in this complex tissue. CD49f and SSEA4 are expressed in SCAPs grown at low oxygen concentration, a condition which increases cell proliferation and stemness [13,54,55]. However, only a minor cell population stains positive for these markers in the native tissue while after isolation and a few divisions in vitro, 80% to 100% of the derived SCAPs expresses the MSC markers (CD90, CD105) along with stemness markers (CD49f and SSEA4) whose expression is O_2_-sensitive. This shows that such a tissue, which contains mainly quiescent cells (very few Ki-67 positive cells), ensures the survival and plasticity of stem cells beside a majority of fibroblast-like cells not yet characterized. Previous studies showed perivascular staining of CXCR4 and Fap alpha. These markers are potentially reflecting the transmigration phenotype of SCAPs into the root channel during apexification [10,11]. Additionally, we detected podosome-like structures, which are actin-rich membrane organelles dedicated to cell invasion by proteolysis. Indeed, the co-expression and colocalization of Tks5 and Cortactin, in the perivascular regions or at the edges of the sections, reveal podosome-like structures suggestive of migrating cells. Interestingly, we could hypothesize that mechanosensing signaling could rapidly lead to migratory cells involved in the formation of the apex of the roots since essential ingredients of podosomes are present within the native tissue [56,57,58,59]. Indeed, Tks5, a mechanosensor protein, is a likely candidate acting as a key scaffolding molecule in podosome forming cells. It recruits different proteins like AFAP-110, the isoform A of p190RhoGAP, and Cortactin, a well-known Src substrate, to sites of podosome formation [59,60]. It will be very interesting to characterize expression of these proteins in cultured SCAPs and to determine whether oxygen concentration regulates the formation of active podosomes towards migration purposes. Also, the present detailed cartography of healthy apical papilla tissue could help to determine whether the structure of this tissue or of dental pulp tissue is altered in pathologies like Molar Incisor Hypomineralization (MIH), a serious health problem which is increasing for still unknown reasons among teenagers [61,62,63].

We previously described SCAP banks isolated and amplified, from the same donor, under atmospheric (21%) or physioxic oxygen concentration (3%) and showed a proliferative advantage of banks isolated and amplified at 3% O_2_ [13]. In this study we described six new banks (UBx-SCAP-N4-N6 and UBx-SCAP-H4-H6) which display similar properties with very stable phenotypes after several freeze/thaw cycles. The improvement of MSCs regenerative properties when amplification is achieved under low oxygen concentration is now well accepted and it seems that only sudden hypoxic/hyperoxic shocks are deleterious in vitro, leading to alterations of MSCs properties or even cell death [44,49,50,51]. For example, short exposure of human bone marrow-derived MSCs to 1% O_2_ decrease their proliferation and differentiation potential without affecting survival [64,65,66]. However, SCAPs overexpressing ephrinB2 secreted more VEGF under hypoxia and stimulated tube formation by endothelial cells in co-culture [67]. Likewise, 5% O_2_ tension enhances the competence of cardiac stem cells (CSC) with an increased proliferation rate [68]. The impact of oxygen levels on cell physiology depends on the duration, concentration of oxygen used, and cell models.

We have investigated the potential involvement of telomerase activity in the proliferative advantage of SCAPs grown under low O_2_ concentration. We found that despite the fact that this enzyme has similar activity in hiPSC, a paradigm of highly proliferative cells, at both O_2_ concentrations, the telomere length is probably not regulated by the sole telomerase activity in SCAPs. Indeed, there is a slight shortening of telomere length over time while telomerase activity was not decreased. Our results are in agreement with a report showing no differences in telomerase activity nor in telomere lengths in Adipose Derived Stem Cells (ADSC) amplified under low O_2_ concentration [54], but is in contrast with the report on cardiac stem cells showing higher telomerase activity and telomere length when cultured at 5% O_2_ in comparison with 21% O_2_ [68]. It should be noted that another mechanism, independent of telomerase activity, has recently been described for telomere lengthening, named Alternative Telomere Lengthening (ALT), in embryos and embryonic stem cells. New players involved in ALT have been identified, such as DDB1 And CUL4 Associated Factor 11 (Dcaf11) and its downstream target, Zscan4 [69,70,71]. Transient expression of Zscan4 promotes telomere recombination, leading to telomere elongation [28,70,71,72]. The ALT pathway deserves to be explored in adult stem cells. Finally, the benefit of low oxygen concentration on SCAP proliferation and differentiation could be a consequence of its effects on the regulation of reactive oxygen species [73]. Indeed, the PI3K/Akt pathway is activated in response to hypoxia and inhibits oxidative stress in a ROS-dependent manner [74]. These new pathways linking oxygen concentration, proliferation and metabolic analyses will deserve future investigations.

SCAPs grown in specific scaffolds in 3D could be relevant devices for regenerative medicine purposes. The hypoxic stress that cells encounter when added to biomaterials and grafted into tissues could be overcome by preconditioning the cells under low oxygenation. Alternatively, providing oxygen to the grafted tissue could be pertinent. For example, it has been shown that in situ oxygenation of SCAPs embedded in a hydrogel like GelMa in the presence of CAO_2_ results in improved graft survival [75]. In the same vein, in situ oxygen generation by oxygen-producing scaffolds could produce and deliver oxygen to the adjacent cells independently of blood perfusion. Specific hydrogels with high oxygen preservation capability were designed to allow MSCs grown at 1% O_2_ to survive for 14 days [76]. In our study, we tested the impact of preconditioning of SCAPs isolated and amplified at 3% O_2_ for their survival in a model of subcutaneous engraftment in immunodeficient mice. We choose a commercial ECM-enriched thixotropic hydrogel in which cells survive in vitro for at least one week (data not shown). This gel is convenient to handle and inject sub-cutaneously because of its thixotropic property. As it gels at 37 °C, the injected cellularized graft does not disperse in the implanted tissue. We observed the preservation of the hydrogel during the 44 days of experiment and a good biocompatibility over time. Unexpectedly, we observed that cells preconditioned at 3% O_2_ survived slightly less than their counterpart at 21%, with a statistical difference up to day 11. However, the protective effect of the hydrogel was observed in both conditions with a stabilization of cell survival after 21 days, with a slightly better effect for cells amplified at 3% O_2_. We observed that cells do not divide and lose their MSC properties within the hydrogel indicating that they could have differentiated to fibroblastic-like cells and that they were not triggered to proliferate since no stress signals was included in our engraftment strategy. Various studies have reported the benefits of hypoxia and cell priming with growth factors to enhance bone defect repair in a mouse calvaria defect model [3,4,77,78,79]. In these studies, angiogenesis and mineralization were improved by short priming at 1% O_2_. However, the remaining dental stem cells after the period of engraftment were not characterized [3,4,77,78,79]. Efficient bone repair was however clearly observed, which might depend on early and efficient paracrine effect of low O_2_ or FGF2- primed cells which might attract endogenous MSCs for bone regeneration.

## 5. Conclusions

In this study we analyzed the native tissue from which the SCAPs are isolated and provide the localization of stem cell niches and identification of podosome-like structures potentially associated with cell migration. We challenged the survival benefit of physioxic preconditioning of SCAPs and demonstrate that while improvements in regeneration in vivo are unexpectedly associated with increased survival only to a limited extent, the real benefit of low O_2_ preconditioning stands in the much higher proliferative capacity of cells which could be amplified for more passages, in vitro, before cell implantation. In addition, in vitro amplification under low oxygen tension conditions could also facilitate their post-implantation adaptation before vascularization of the graft.

## Data Availability

Not applicable.

## Supplementary Materials

The following supporting information can be downloaded at: https://www.mdpi.com/article/10.3390/cells11244098/s1, Figure S1: ImmunoMap of apical papilla sections. Figure S2: Tracking of common labeling areas on adjacent sections of apical papilla. Figure S3: Stable higher expression of CD49f and SSEA4 at 3% versus 21% O2. Figure S4: Detailed descriptions and statistical analyses in complement of Figure 6. Figure S5: Distribution of the measurement shown as Boxplot to visualize the scattering of the data for all mice on each day of measure.
