# Supplementary material for: The Journey of SCAPs (Stem Cells from Apical Papilla), from Their Native Tissue to Grafting: Impact of Oxygen Concentration
Source: Cells. 2022 Dec 16;11(24):4098. doi: 10.3390/cells11244098 (PMC9776846; doi:10.3390/cells11244098)

Supplementary Materials

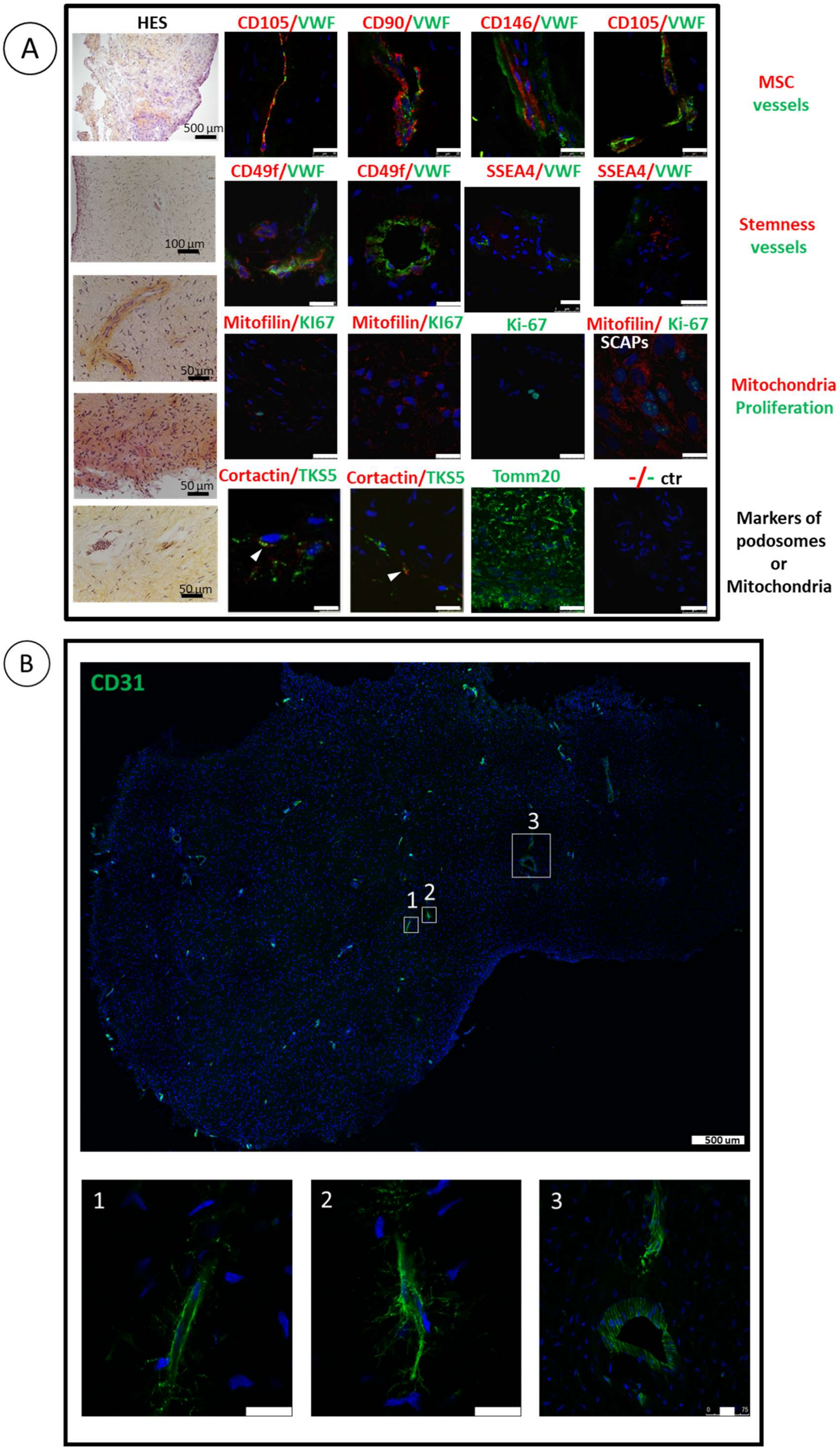

C

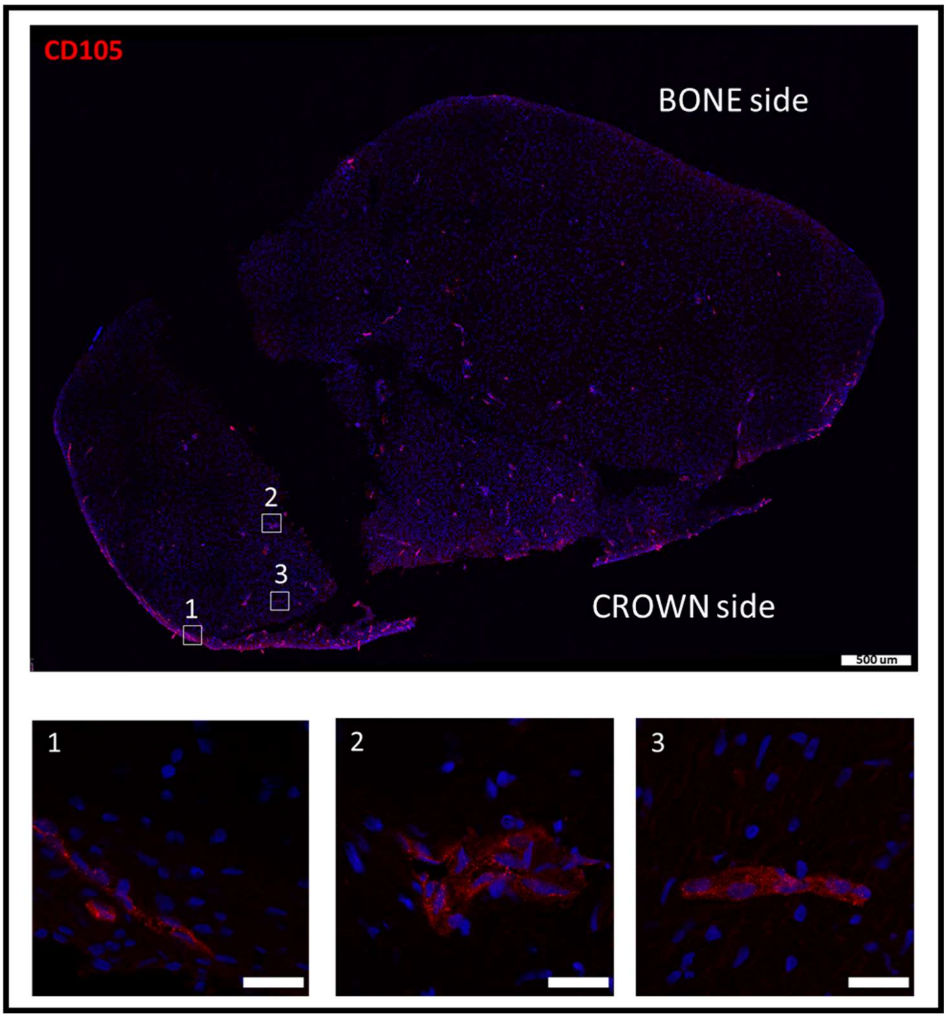
CD105
BONE side
CROWN side
1
2
3
500 um
1
2
3

D

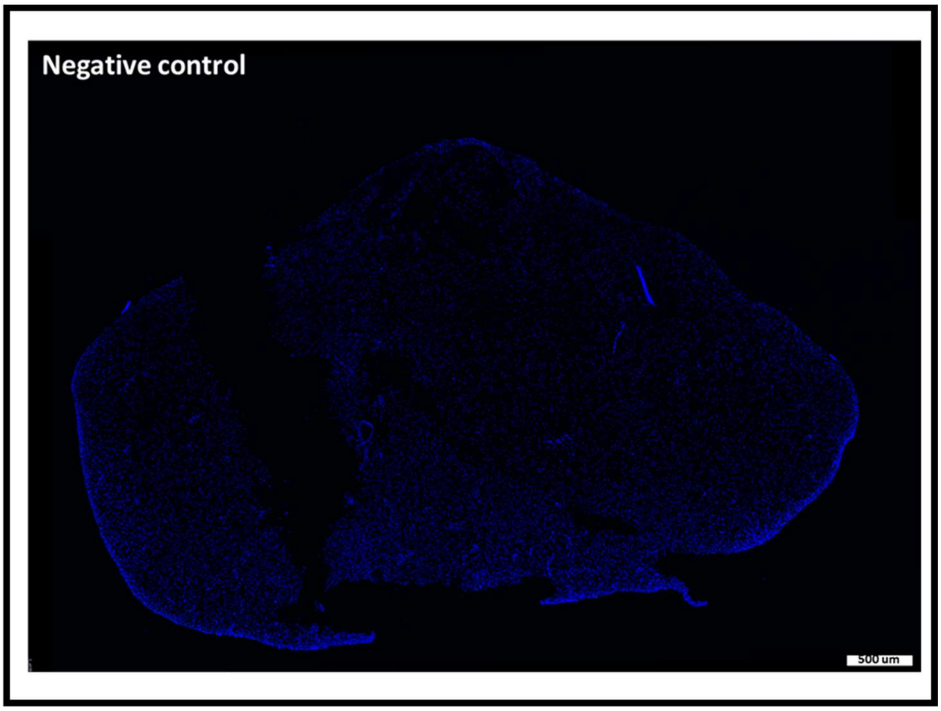
Negative control
500 um

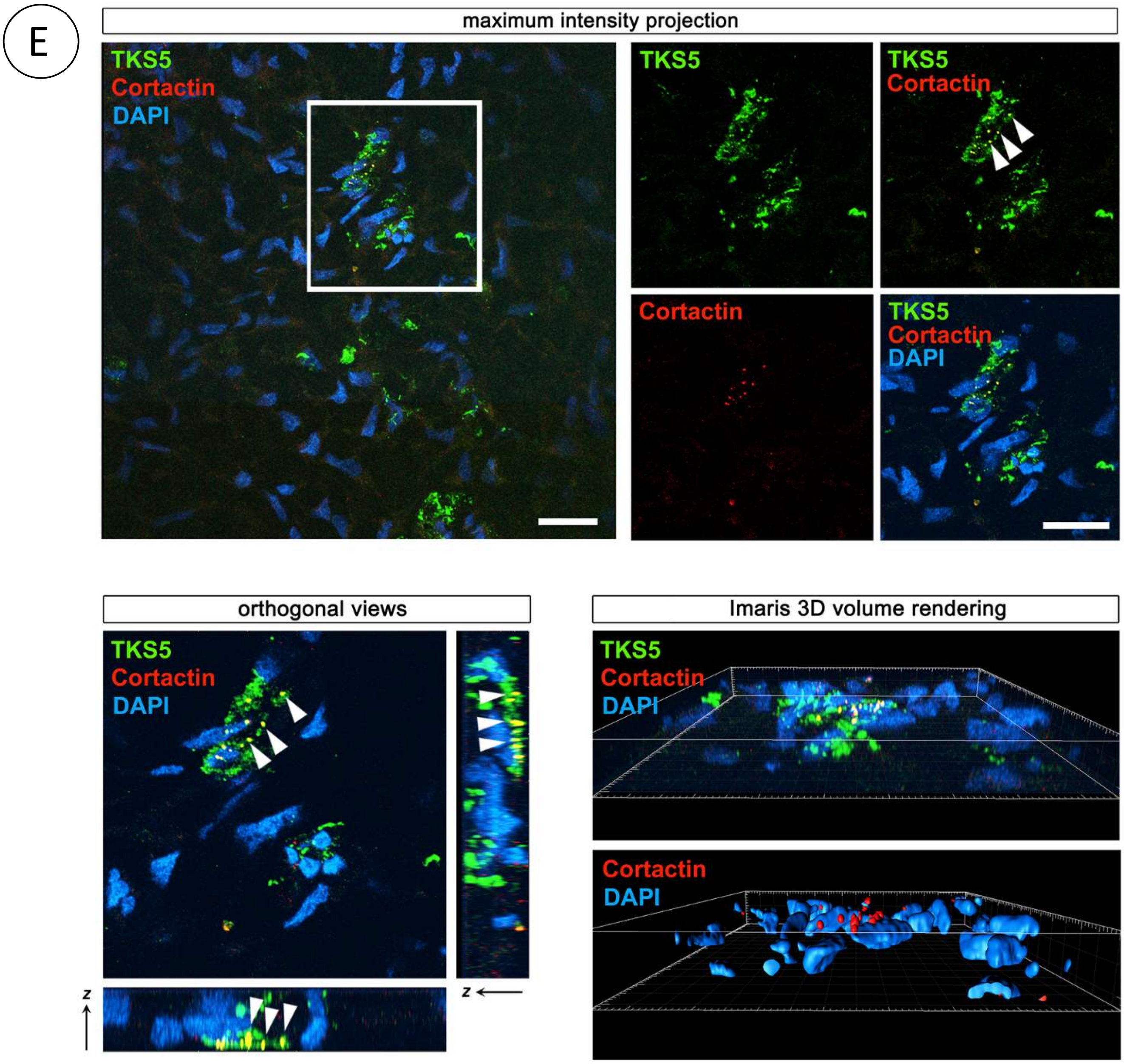

Figure S1: ImmunoMap of apical papilla sections. **A)** Additive pictures of 10µm apical papilla sections stained with HES or immunolabelled with antibodies as indicated, in complement of Figure 1. These pictures correspond to representative images of experiments performed on at least 5 independent blocs of apical papilla derived for 5 donors (teenager, boys, 15 year old). Scale bars are indicated on each HES pictures and are 25 µm for immunolabelling except for the left double Cortactin/ TKS5 staining, image: 10 µm. White arrows indicate the Tks5/Cortactin co-labeling. Nucleus were stained with DAPI. Negative control (-/- ctr) is incubation of the section with secondary antibodies only. A positive control for mitofilin and Ki-67 has been performed with SCAPs cells grown under 3% O_2, 4 days: (SCAPs). **B)** Immunolabelling of the entire section for CD31 and enlargement of three areas as indicated. **C)** Immunolabelling of the entire section with CD105 antibody and enlargement of three areas as indicated. **D)** Immunolabelling of the entire section with secondary antibodies only. Scale bar of entire section is 500 µm and scale bar of the enlargement is 25 µm. All sections were counterstained with DAPI for nuclei visualization. **E)** Upper panels: Representative images of an apical papilla tissue section double stained for the podosome markers TKS5 (green) and Cortactin (red). Nuclei are visualized with DAPI (blue) (left

panel). High magnification image of the boxed region showing the individual and merged channels highlights the colocalization of the two different podosome components (right panels). Scale bars, 20 μm. Lower panels: Orthogonal views of the z-stack used to generate the picture shown in upper panels reveal that colocalized TKS5 and Cortactin signals are detected at a higher focal plane compared to DAPI, (left panels). 3D volume reconstructions of the TKS5 (green), Cortactin (red) and DAPI (blue) signals from the very same area (right panels). White arrows indicate the Tks5/ Cortactin co-labeling.

Supplemental data 2A

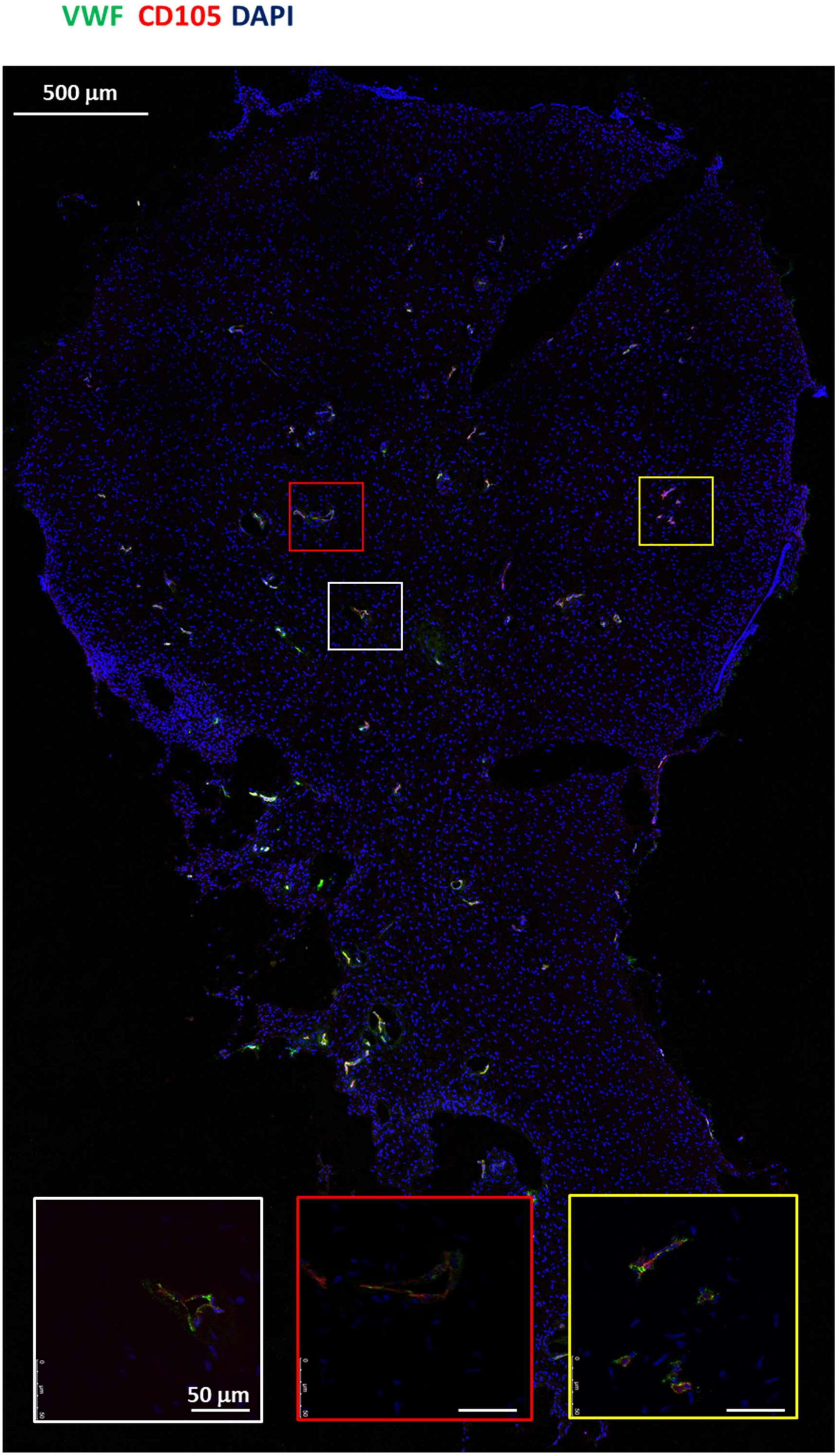

Supplemental data 2B

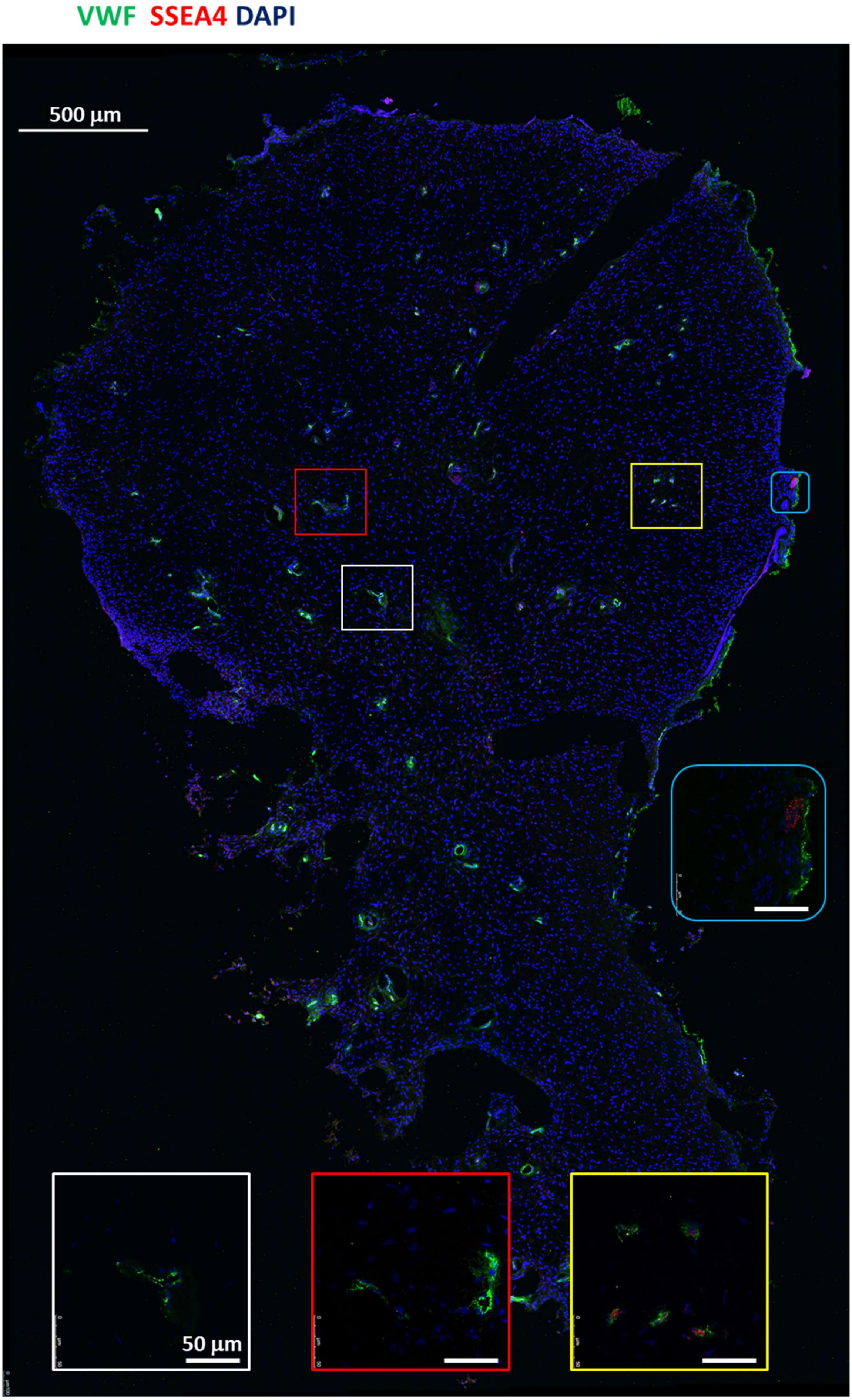

Supplemental data 2C

VWF CD49f DAPI

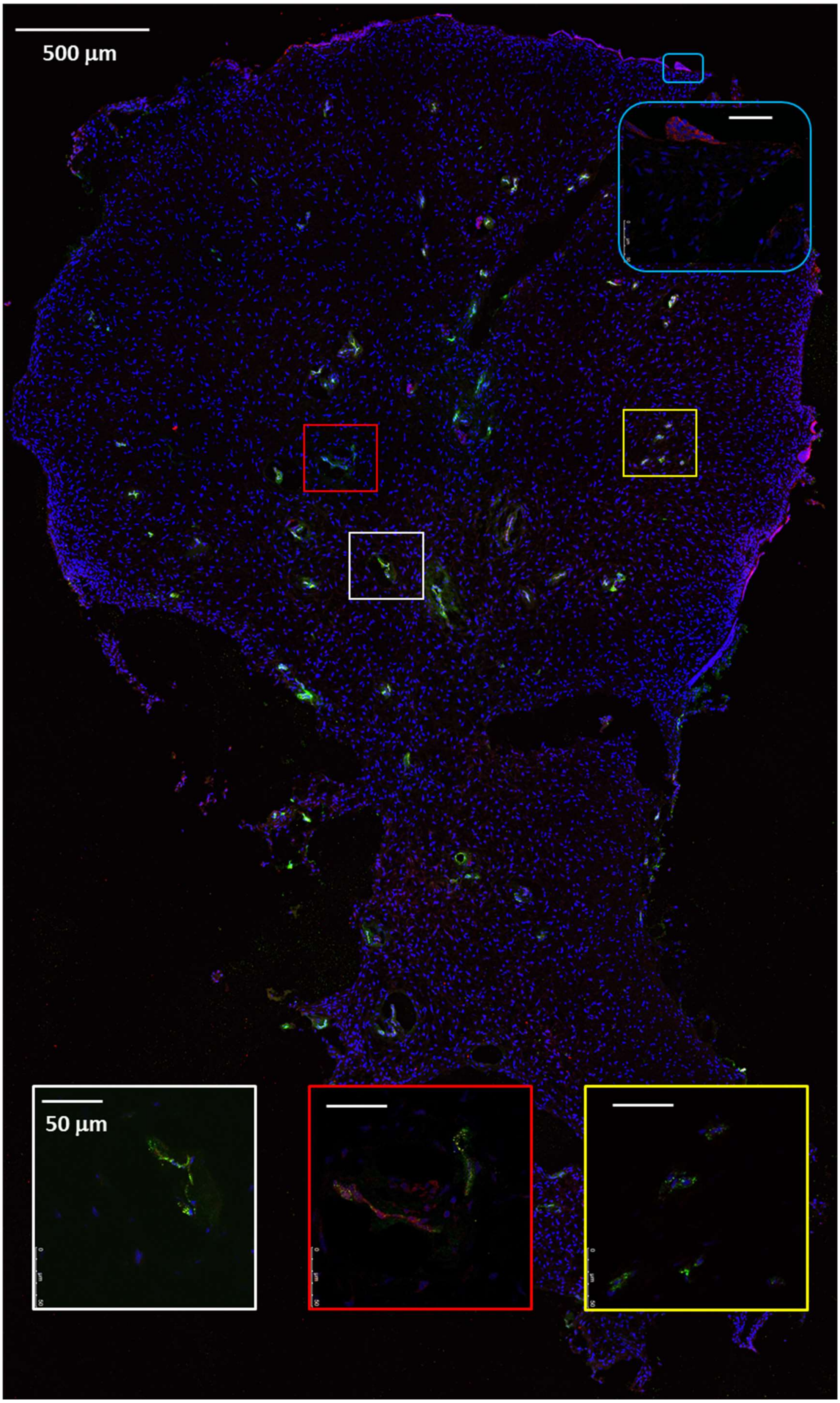

Supplemental data 2D

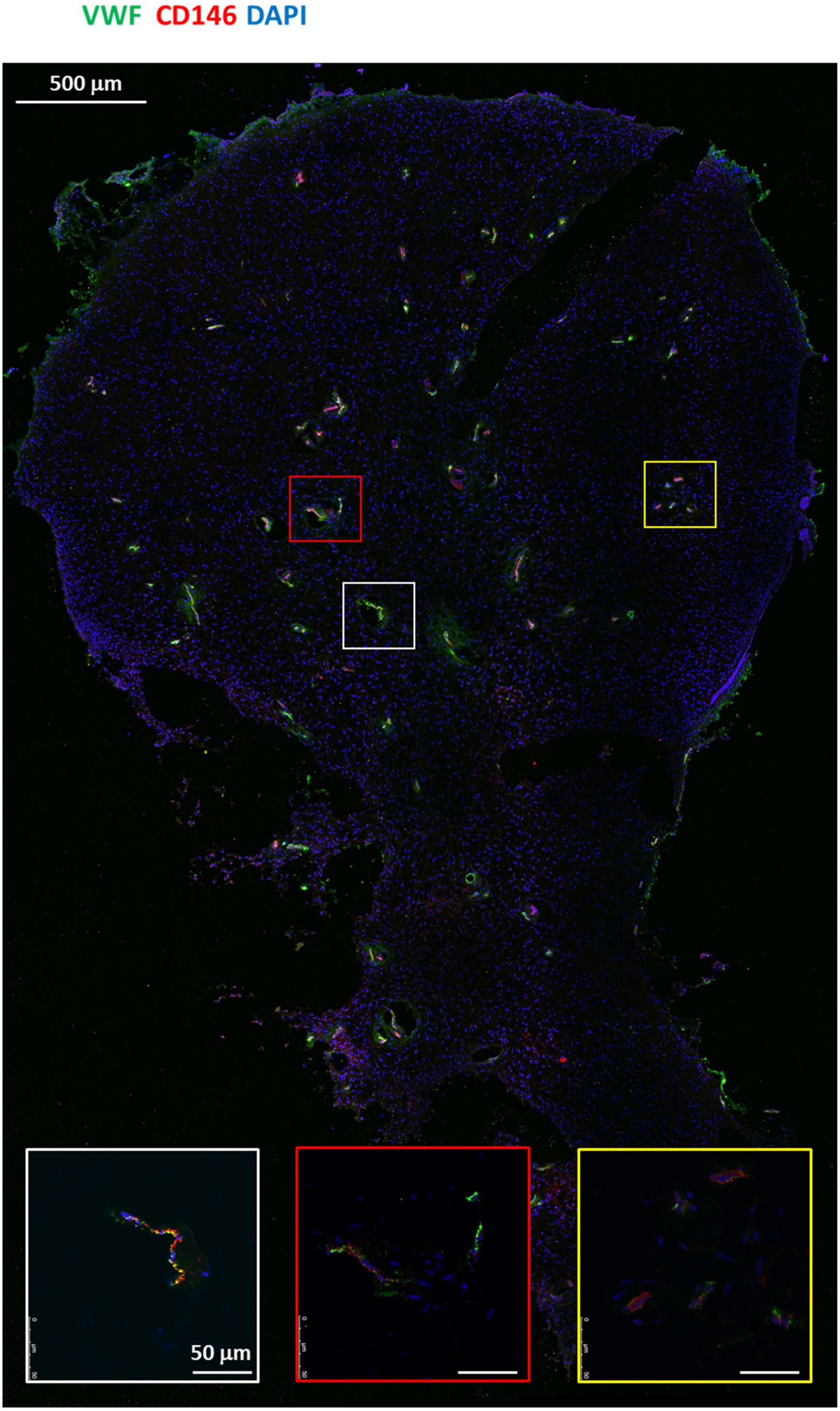

Supplemental data 2E

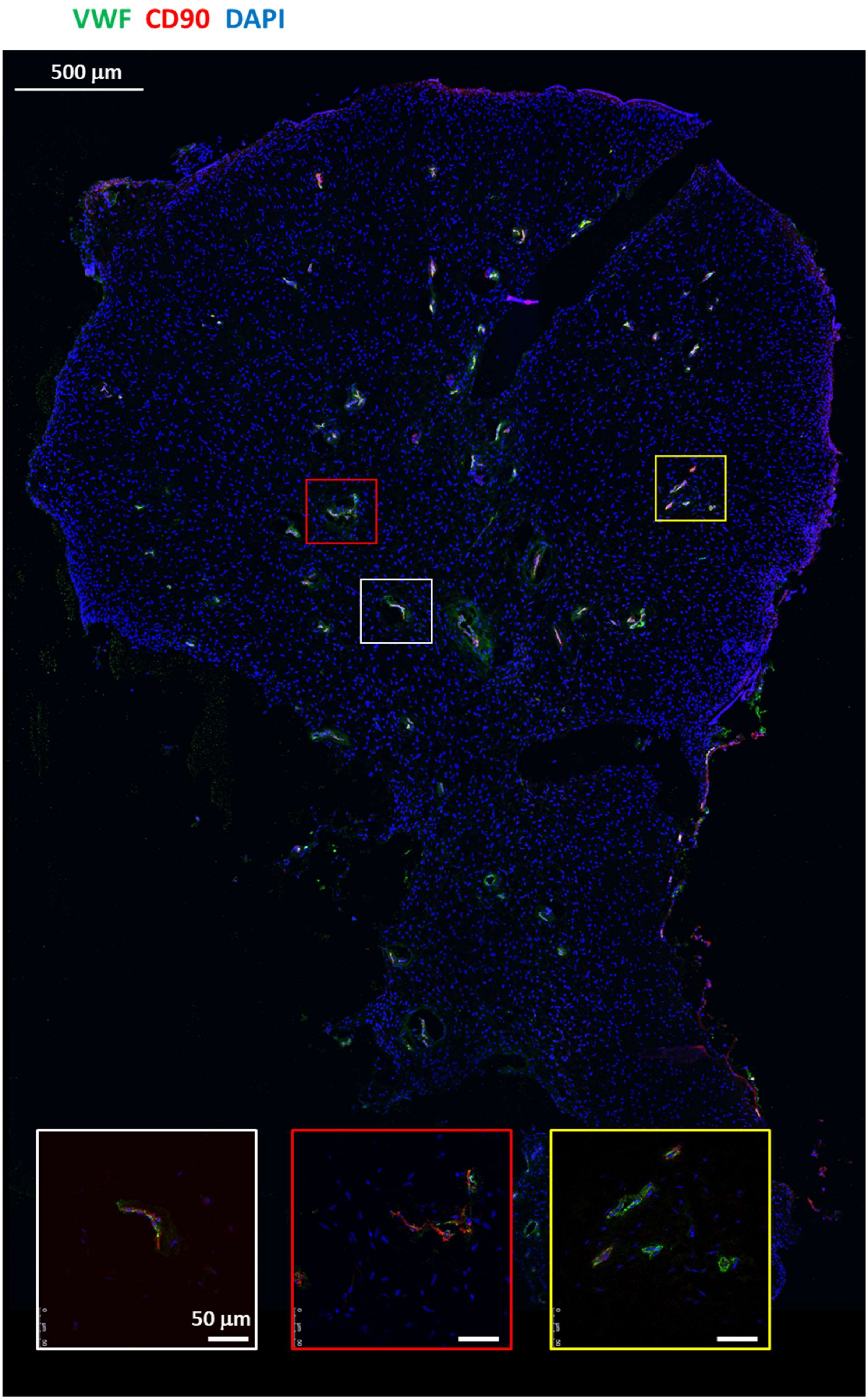

Supplemental data 2F

Cneg Cneg (Secondary antibodies only)

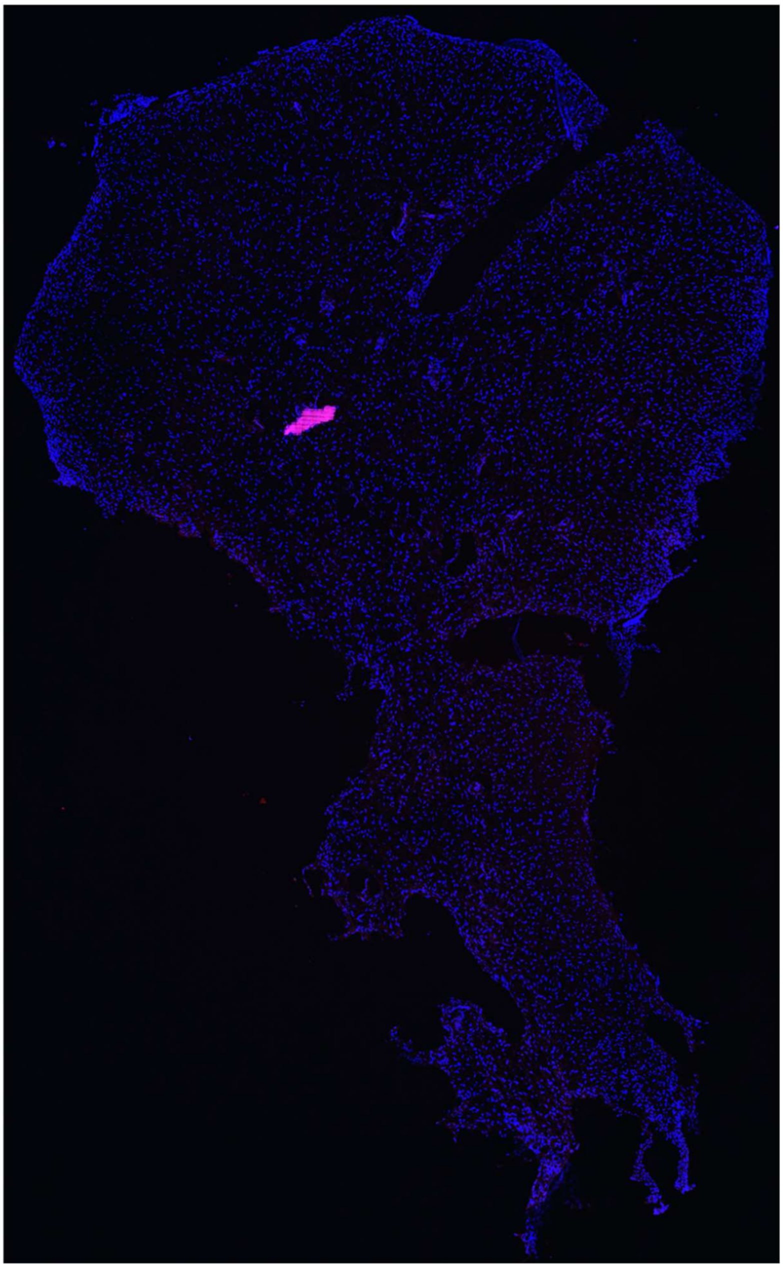

Figure S2: Tracking of common labeling areas on adjacent sections of apical papilla.
Each section was labeled separately with the different antibodies as indicated (CD90, CD105, CD146, CD49f, SSEA4) and, for all sections, with the anti-Von Willebrand Factor antibody (VWF), which labels the blood vessels and allowed to have common reference areas on the different sections. Analyses and pictures recording were then performed with the confocal Leica microscope: for each section, a mosaic of 40 pictures was made with the 10x objective (VWF in green and the other marker in red) to have the complete picture of each section. We then selected 3 similar areas on each section and then take pictures at the 40x objective which are presented as colored squares, at a higher magnification.
The MSC markers (CD90, CD105 and CD146) were detected close or in very similar areas as blood vessels. For SSEA4 and CD49f, some areas were labelled at proximity of the blood vessels and MSC containing areas but staining away from vessels and from the MSC markers were also observed (as shown with the enlarged area).

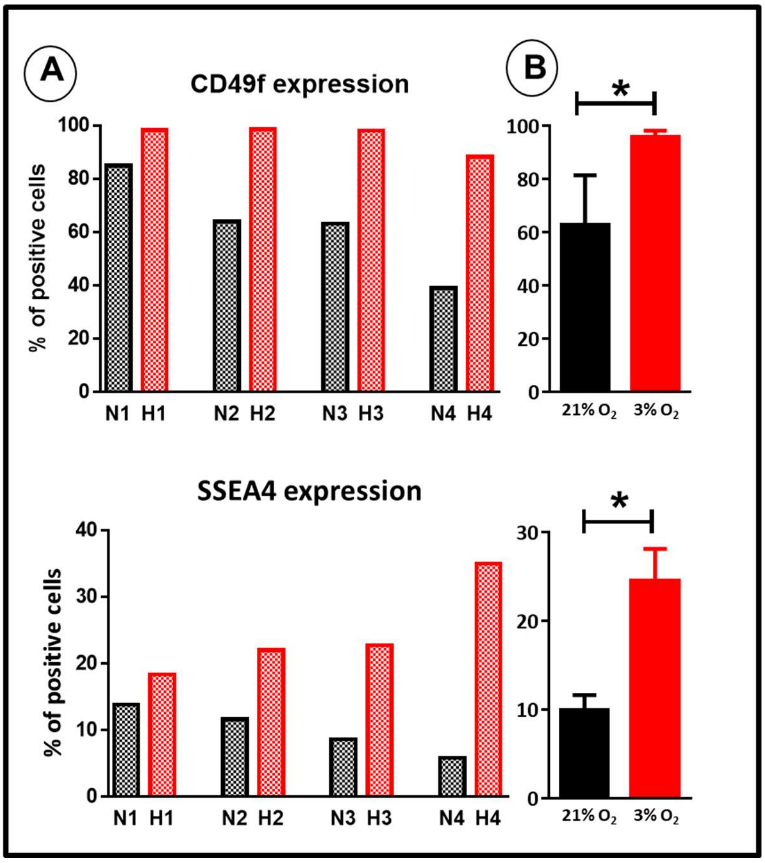

Figure S3: Stable higher expression of CD49f and SSEA4 at 3% versus 21% O_2. **A)** Graphs represent the percentage of positive cells obtained with the various banks freezed and thawed several time (at least 3 cycles), analysed by flow cytometry **B)** Mean + SD of the 4 banks (Mann-Whitney U, *: p-value<0.05).

Figure S4: Detailed descriptions and statistical analyses in complement of Figure 6.

Results on normalized data from day 1 and day by day from which the graphs shown in Figure 6B were drawn (mean+ Std. error mean).
Descriptives and Statistiques

1) Descriptives

	O2	Gel	D1	D4	D7	D11	D14	D17	D21	D28	D44
N	Normoxia	Without gel	10	10	10	10	10	10	10	7	7
		With gel	10	10	10	10	10	10	10	7	7
	Hypoxia	Without gel	10	10	10	10	10	10	10	7	7
		With gel	10	10	10	10	10	10	10	7	7
Missing	Normoxia	Without gel	0	0	0	0	0	0	0	3	3
		With gel	0	0	0	0	0	0	0	3	3
	Hypoxia	Without gel	0	0	0	0	0	0	0	3	3
		With gel	0	0	0	0	0	0	0	3	3
Mean	Normoxia	Without gel	1.00	1.03	1.43	1.02	0.817	1.19	1.15	0.787	0.916
		With gel	1.00	2.02	3.96	5.14	5.54	9.15	19.6	9.78	29.3
	Hypoxia	Without gel	1.00	0.533	0.621	0.364	0.479	0.764	1.11	0.767	1.32
		With gel	1.00	2.84	3.90	7.21	9.11	14.7	39.9	14.1	65.4
Std. error mean	Normoxia	Without gel	0.00	0.192	0.220	0.125	0.143	0.287	0.187	0.111	0.112
		With gel	0.00	0.273	0.509	0.681	1.67	1.81	4.49	3.13	10.0
	Hypoxia	Without gel	0.00	0.0549	0.0839	0.0425	0.0742	0.144	0.209	0.175	0.235
		With gel	0.00	0.582	0.561	1.54	1.78	3.36	8.37	3.24	17.8
Median	Normoxia	Without gel	1.00	0.950	1.23	1.10	0.655	0.985	1.02	0.930	1.00
		With gel	1.00	1.71	3.75	4.89	3.67	8.64	15.2	6.36	24.2
	Hypoxia	Without gel	1.00	0.540	0.545	0.360	0.465	0.610	0.775	0.720	1.53
		With gel	1.00	2.25	3.83	6.04	7.93	11.0	29.8	11.4	66.4
Standard deviation	Normoxia	Without gel	0.00	0.606	0.694	0.396	0.451	0.909	0.592	0.293	0.297
		With gel	0.00	0.865	1.61	2.15	5.27	5.74	14.2	8.29	26.6
	Hypoxia	Without gel	0.00	0.174	0.265	0.134	0.235	0.456	0.660	0.463	0.623
		With gel	0.00	1.84	1.77	4.88	5.64	10.6	26.5	8.57	47.2
Minimum	Normoxia	Without gel	1.00	0.470	0.510	0.310	0.370	0.470	0.320	0.370	0.470
		With gel	1.00	0.830	1.54	2.71	1.50	1.51	1.18	3.35	5.53
	Hypoxia	Without gel	1.00	0.250	0.380	0.160	0.160	0.300	0.430	0.260	0.400
		With gel	1.00	0.650	1.18	2.48	3.58	3.54	11.9	4.76	19.4
Maximum	Normoxia	Without gel	1.00	2.56	2.42	1.56	1.75	3.62	2.21	1.06	1.37
		With gel	1.00	3.72	6.50	8.27	19.8	18.6	42.2	27.4	85.2
	Hypoxia	Without gel	1.00	0.790	1.25	0.530	0.990	1.73	2.11	1.70	2.30
		With gel	1.00	6.45	6.72	16.6	17.9	36.5	78.5	27.6	142

1) Descriptives

	O2	Gel	D1	D4	D7	D11	D14	D17	D21	D28	D44
Shapiro-Wilk W	Normoxia	Without gel	NaN	0.788	0.916	0.950	0.878	0.694	0.944	0.831	0.974
		With gel	NaN	0.945	0.967	0.890	0.654	0.949	0.912	0.761	0.800
	Hypoxia	Without gel	NaN	0.969	0.807	0.929	0.925	0.869	0.839	0.874	0.961
		With gel	NaN	0.928	0.960	0.869	0.857	0.896	0.866	0.877	0.892
Shapiro-Wilk p	Normoxia	Without gel	NaN	0.010	0.326	0.663	0.125	< .001	0.602	0.082	0.927
		With gel	NaN	0.615	0.857	0.169	< .001	0.653	0.297	0.017	0.041
	Hypoxia	Without gel	NaN	0.882	0.017	0.440	0.404	0.098	0.043	0.199	0.824
		With gel	NaN	0.427	0.785	0.097	0.069	0.200	0.090	0.214	0.284

2) Statistiques

With vs without gel in 21% O_2 – Mann-Whitney U (Non-parametric)

Independent Samples T-Test

		Statistic	p
D4	Mann-Whitney U	13.00	0.004
D7	Mann-Whitney U	4.00	< .001
D11	Mann-Whitney U	0.00	< .001
D14	Mann-Whitney U	1.00	< .001
D17	Mann-Whitney U	2.00	< .001
D21	Mann-Whitney U	3.00	< .001
D28	Mann-Whitney U	0.00	< .001
D44	Mann-Whitney U	0.00	< .001

With vs without gel in 3% O_2 – Mann-Whitney U (Non-parametric)

Independent Samples T-Test

		Statistic	p
D4	Mann-Whitney U	3.00	< .001
D7	Mann-Whitney U	1.00	< .001
D11	Mann-Whitney U	0.00	< .001
D14	Mann-Whitney U	0.00	< .001
D17	Mann-Whitney U	0.00	< .001
D21	Mann-Whitney U	0.00	< .001
D28	Mann-Whitney U	0.00	< .001
D44	Mann-Whitney U	0.00	< .001

Non parametric paired Samples test

Paired Samples T-Test

			Statistic	p
D4_Normoxia_No gel	D4_Normoxia_Gel	Wilcoxon W	2.00	0.006
D4_Hypoxia_No gel	D4_Hypoxia_Gel	Wilcoxon W	1.00	0.004
D4_Normoxia_No gel	D4_Hypoxia_No gel	Wilcoxon W	52.00	0.010
D4_Normoxia_Gel	D4_Hypoxia_Gel	Wilcoxon W	17.00	0.322
D7_Normoxia_No gel	D7_Normoxia_Gel	Wilcoxon W	0.00	0.006
D7_Hypoxia_No gel	D7_Hypoxia_Gel	Wilcoxon W	0.00	0.002
D7_Normoxia_No gel	D7_Hypoxia_No gel	Wilcoxon W	50.00	0.020
D7_Normoxia_Gel	D7_Hypoxia_Gel	Wilcoxon W	29.00	0.922
D11_Normoxia_No gel	D11_Normoxia_Gel	Wilcoxon W	0.00	0.002
D11_Hypoxia_No gel	D11_Hypoxia_Gel	Wilcoxon W	0.00	0.002
D11_Normoxia_No gel	D11_Hypoxia_No gel	Wilcoxon W	54.00	0.004
D11_Normoxia_Gel	D11_Hypoxia_Gel	Wilcoxon W	15.00	0.232
D14_Normoxia_No gel	D14_Normoxia_Gel	Wilcoxon W	0.00	0.002
D14_Hypoxia_No gel	D14_Hypoxia_Gel	Wilcoxon W	0.00	0.002
D14_Normoxia_No gel	D14_Hypoxia_No gel	Wilcoxon W	46.00	0.066
D14_Normoxia_Gel	D14_Hypoxia_Gel	Wilcoxon W	15.00	0.232
D17_Normoxia_No gel	D17_Normoxia_Gel	Wilcoxon W	0.00	0.006
D17_Hypoxia_No gel	D17_Hypoxia_Gel	Wilcoxon W	0.00	0.002
D17_Normoxia_No gel	D17_Hypoxia_No gel	Wilcoxon W	42.50	0.139
D17_Normoxia_Gel	D17_Hypoxia_Gel	Wilcoxon W	17.00	0.322
D21_Normoxia_No gel	D21_Normoxia_Gel	Wilcoxon W	0.00	0.002
D21_Hypoxia_No gel	D21_Hypoxia_Gel	Wilcoxon W	0.00	0.002
D21_Normoxia_No gel	D21_Hypoxia_No gel	Wilcoxon W	28.00	1.000
D21_Normoxia_Gel	D21_Hypoxia_Gel	Wilcoxon W	10.00	0.084
D28_Normoxia_No gel	D28_Normoxia_Gel	Wilcoxon W	0.00	0.063
D28_Hypoxia_No gel	D28_Hypoxia_Gel	Wilcoxon W	0.00	0.063
D28_Normoxia_No gel	D28_Hypoxia_No gel	Wilcoxon W	7.00	1.000
D28_Normoxia_Gel	D28_Hypoxia_Gel	Wilcoxon W	4.00	0.438
D44_Normoxia_No gel	D44_Normoxia_Gel	Wilcoxon W	0.00	0.063
D44_Hypoxia_No gel	D44_Hypoxia_Gel	Wilcoxon W	0.00	0.063
D44_Normoxia_No gel	D44_Hypoxia_No gel	Wilcoxon W	0.00	0.063
D44_Normoxia_Gel	D44_Hypoxia_Gel	Wilcoxon W	2.00	0.188

Figure S5: Distribution of the measurement shown as Boxplot to visualize the scattering of the data for all mice on each day of measures.

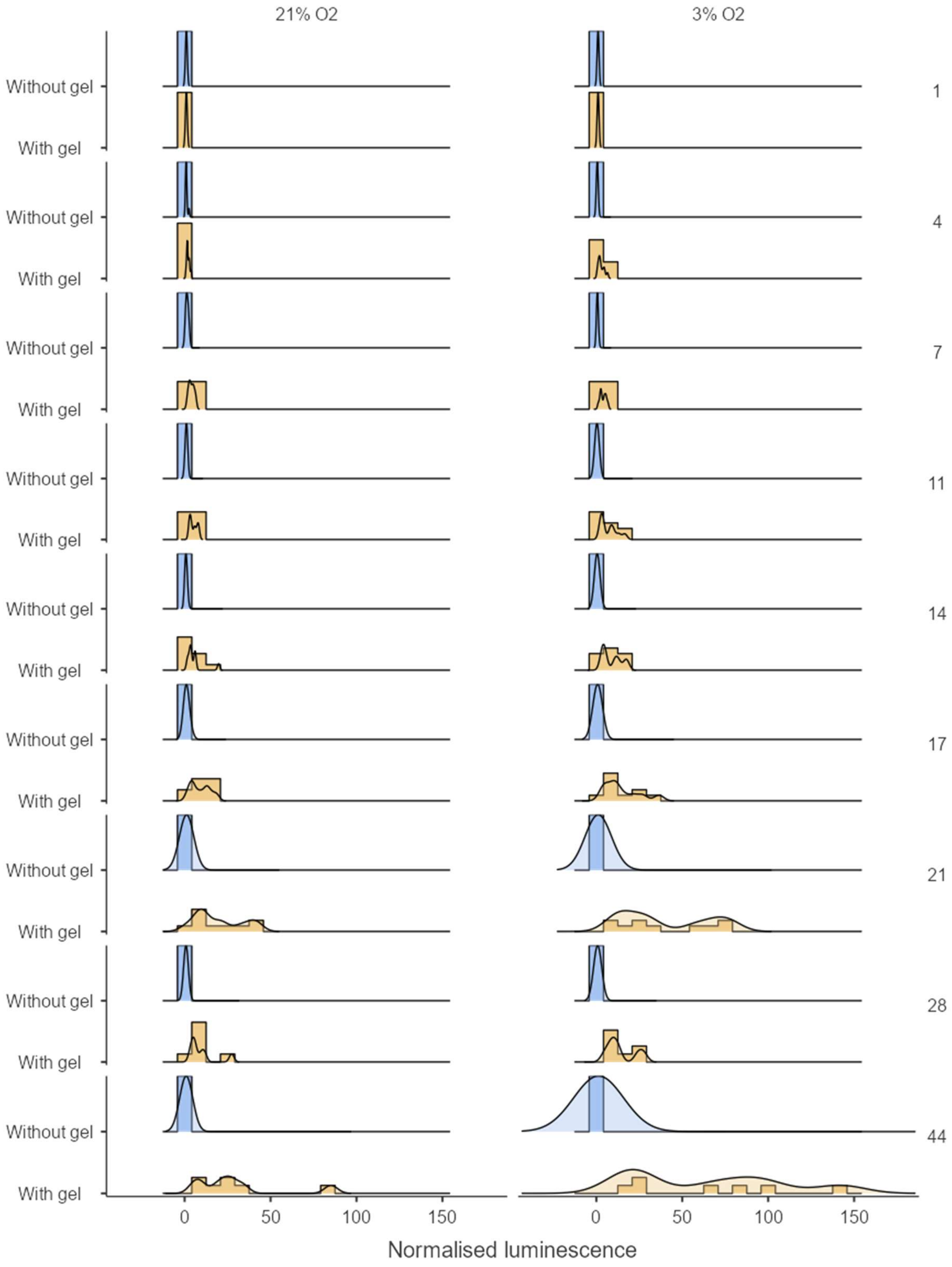

Supplement: Supplementary file 1 [file cells-11-04098-s001.zip › cells-1947706-supplementary.pdf]
